# Molecular Mechanisms for Drug Hypersensitivity Induced by the Malaria Parasite’s Chloroquine Resistance Transporter

**DOI:** 10.1371/journal.ppat.1005725

**Published:** 2016-07-21

**Authors:** Sashika N. Richards, Megan N. Nash, Eileen S. Baker, Michael W. Webster, Adele M. Lehane, Sarah H. Shafik, Rowena E. Martin

**Affiliations:** Research School of Biology, Australian National University, Canberra, Australia; Drexel University College of Medicine, UNITED STATES

## Abstract

Mutations in the *Plasmodium falciparum* ‘chloroquine resistance transporter’ (PfCRT) confer resistance to chloroquine (CQ) and related antimalarials by enabling the protein to transport these drugs away from their targets within the parasite’s digestive vacuole (DV). However, CQ resistance-conferring isoforms of PfCRT (PfCRT^CQR^) also render the parasite hypersensitive to a subset of structurally-diverse pharmacons. Moreover, mutations in PfCRT^CQR^ that suppress the parasite’s hypersensitivity to these molecules simultaneously reinstate its sensitivity to CQ and related drugs. We sought to understand these phenomena by characterizing the functions of PfCRT^CQR^ isoforms that cause the parasite to become hypersensitive to the antimalarial quinine or the antiviral amantadine. We achieved this by measuring the abilities of these proteins to transport CQ, quinine, and amantadine when expressed in *Xenopus* oocytes and complemented this work with assays that detect the drug transport activity of PfCRT in its native environment within the parasite. Here we describe two mechanistic explanations for PfCRT-induced drug hypersensitivity. First, we show that quinine, which normally accumulates inside the DV and therewithin exerts its antimalarial effect, binds extremely tightly to the substrate-binding site of certain isoforms of PfCRT^CQR^. By doing so it likely blocks the normal physiological function of the protein, which is essential for the parasite’s survival, and the drug thereby gains an additional killing effect. In the second scenario, we show that although amantadine also sequesters within the DV, the parasite’s hypersensitivity to this drug arises from the PfCRT^CQR^-mediated transport of amantadine from the DV into the cytosol, where it can better access its antimalarial target. In both cases, the mutations that suppress hypersensitivity also abrogate the ability of PfCRT^CQR^ to transport CQ, thus explaining why rescue from hypersensitivity restores the parasite’s sensitivity to this antimalarial. These insights provide a foundation for understanding clinically-relevant observations of inverse drug susceptibilities in the malaria parasite.

## Author Summary

In acquiring resistance to one drug, many pathogens and cancer cells become hypersensitive to other drugs. This phenomenon could be exploited to combat existing drug resistance and to delay the emergence of resistance to new drugs. However, much remains to be understood about the mechanisms that underlie drug hypersensitivity in otherwise drug-resistant microbes. Here, we describe two mechanisms by which the *Plasmodium falciparum* ‘chloroquine resistance transporter’ (PfCRT) causes the malaria parasite to become hypersensitive to structurally-diverse drugs. First, we show that an antimalarial drug that normally exerts its killing effect within the parasite’s digestive vacuole is also able to bind extremely tightly to certain forms of PfCRT. This activity will block the natural, essential function of the protein and thereby provide the drug with an additional killing effect. The second mechanism arises when a cytosolic-acting drug that normally sequesters within the digestive vacuole is leaked back into the cytosol via PfCRT. In both cases, mutations that suppress hypersensitivity also abrogate the ability of PfCRT to transport chloroquine, thus explaining why rescue from hypersensitivity restores the parasite’s sensitivity to this antimalarial. These insights provide a foundation for understanding and exploiting the hypersensitivity of chloroquine-resistant parasites to several of the current antimalarials.

## Introduction

Originally identified as the protein responsible for conferring resistance to the ‘wonder-drug’ chloroquine (CQ) [1, 2], the *Plasmodium falciparum* ‘chloroquine resistance transporter’ (PfCRT) has become a key player in the malaria parasite’s steadily expanding resistance to drugs [3–5]. The isoforms of PfCRT that confer CQ resistance (PfCRT^CQR^) render the parasite less susceptible to many other compounds [6–10], but also simultaneously induce hypersensitivity to a subset of structurally-diverse molecules [6, 10–18]. This phenomenon, whereby resistance to one drug causes hypersensitivity to another, is known as ‘inverse susceptibility’ or ‘collateral sensitivity’ and has been observed in a wide range of pathogens and cancer cells [19–22]. The growing awareness of the propensity of drug-resistant pathogens to exhibit hypersensitivity to one or more other drugs has sparked interest in the potential for exploiting this Achilles’ heel to combat existing drug resistance and to delay the emergence of resistance to new drugs [19]. However, it is not known how PfCRT^CQR^ isoforms induce hypersensitivity to certain drugs and, more generally, much remains to be understood about the molecular mechanisms that underpin collateral sensitivity in pathogens and cancer cells [20–22].

The ability of PfCRT to affect the activity of so many compounds is likely to be a product of its location at the membrane of the parasite’s digestive vacuole (DV) [1, 11]; an acidic compartment in which many types of antimalarials accumulate and/or act. The parasite takes up hemoglobin and digests it within this compartment in order to grow within its host erythrocyte. This process releases heme monomers that are detoxified via conversion into the inert crystal hemozoin. Quinoline-type antimalarial drugs, including CQ, quinine, and quinidine, concentrate within the DV via ‘weak-base trapping’ [23], where they exert an antimalarial effect by binding to heme and arresting its detoxification [24–27]. Resistance to these quinolines is associated with reductions in the accumulation of the drugs within the DV [8, 11, 28] and we have previously obtained direct evidence of this phenomenon being due, at least in part, to the ability of PfCRT^CQR^ isoforms to efflux CQ, quinine, and quinidine from this compartment. This was achieved by expressing a PfCRT^CQR^ isoform from Dd2 parasites (PfCRT^Dd2^) and the wild-type protein (PfCRT^3D7^) in *Xenopus laevis* oocytes. PfCRT^Dd2^ was found to possess significant CQ, quinine, and quinidine transport activity, whereas PfCRT^3D7^ did not [29–31]. These findings were consistent with a number of biochemical studies that had provided indirect evidence of drug transport via PfCRT^CQR^ isoforms. For example, PfCRT^CQR^ isoforms had been linked to the efflux of CQ and quinine from parasite-infected red blood cells [32, 33], and PfCRT^CQR^ was also implicated in the quinoline-induced efflux of protons from the DV of CQ-resistant parasites [34–36]. Moreover, the expression of PfCRT^Dd2^ at endosomal membranes within *Dictyostelium discoideum* reduced the accumulation of CQ and quinine within these vesicles, consistent with the mutant protein mediating the transport of these two drugs [37, 38]. Aside from modulating the parasite’s susceptibility to diverse pharmacons, PfCRT fulfills an essential [39, 40] but currently unresolved physiological function in the parasite.

In this study, we investigated the mechanistic basis of PfCRT-induced drug hypersensitivity by characterizing the isoforms of PfCRT carried by nine different parasite lines (Table 1). These lines were generated by Cooper, Johnson, and colleagues [11, 12, 14, 15] and were produced by applying CQ, quinine, and/or amantadine pressure to either the CQ-sensitive strain 106/1 or the CQ-resistant strain K1. CQ-resistant strains, including K1, are hypersensitive to amantadine [13, 15]—a weak-base antiviral drug that is thought to accumulate in acidic organelles [41]. Like all PfCRT^CQR^ isoforms identified to date, the K1 variant of PfCRT (PfCRT^K1^) contains a mutation at position 76, where a positively-charged lysine (K) residue is replaced by an uncharged residue (usually threonine; T) (S1 Fig). The K76T mutation is necessary (but not sufficient) to enable PfCRT to mediate the transport of protonated CQ [30, 42]. The version of PfCRT carried by 106/1 retains 76K but is otherwise identical to PfCRT^K1^. Pressuring 106/1 parasites with CQ resulted in the CQ-resistant lines 106/1^76T^, 106/1^76I^, and 106/1^76N^, which carried either PfCRT^K1^ or PfCRT^K1^ with an isoleucine (I) or an asparagine (N) at position 76 (76I-PfCRT^K1^ and 76N-PfCRT^K1^, respectively) [11]. The latter two lines displayed unexpected drug responses: 106/1^76I^ was hypersensitive to quinine and 106/1^76N^ was sensitive to quinidine [11, 12]. The subsequent selection of 106/1^76I^ parasites with quinine resulted in the reintroduction of a positively-charged residue (C72R, Q352K, or Q352R) into 76I-PfCRT^K1^ that suppressed the parasite’s hypersensitivity to quinine but also re-sensitized it to CQ [12]. This ‘reciprocal collateral sensitivity’ was also observed when K1 parasites or the 106/1^76I^ line were pressured with amantadine [14, 15]; the resulting parasites gained mutations in PfCRT (including S163R and V369F), were no longer hypersensitive to amantadine, but were rendered sensitive to CQ.

**Table 1:** The origins, drug susceptibilities, and PfCRT haplotypes of different 106/1 and K1 *P*. *falciparum* lines.

Line or isolate^a^	Origin or drug selection	PfCRT variant	Drug susceptibility^b^ (*In vitro* resistance index)			Amino acid changes in PfCRT^c^											
			CQ	QN	QD	72	74	75	76	163	220	271	326	352	369	356	371
HON	Africa	PfCRT^3D7^	1	1	1	C	M	N	K	S	A	Q	N	Q	V	I	R
Dd2	Indochina/Laos	PfCRT^Dd2^	11.78 ± 0.18	1.82 ± 0.31	2.88 ± 0.46	C	***I***	***E***	***T***	S	***S***	***E***	***S***	Q	V	***T***	***I***
106/1	Sudan	76K-PfCRT^K1^	1.10 ± 0.06	1.08 ± 0.06	1.01 ± 0.01	C	***I***	***E***	K	S	***S***	***E***	***S***	Q	V	I	***I***
106/1^76T^	CQ	PfCRT^K1^	12.95 ± 0.95	1.69 ± 0.19	1.95 ± 0.05	C	***I***	***E***	***T***	S	***S***	***E***	***S***	Q	V	I	***I***
106/1^76N^	CQ	76N-PfCRT^K1^	8.60 ± 0.90	1.56 ± 0.16	1.08 ± 0.08	C	***I***	***E***	***N***	S	***S***	***E***	***S***	Q	V	I	***I***
106/1^76I^	CQ	76I-PfCRT^K1^	15.66 ± 2.20	0.11 ± 0.04	1.72 ± 0.13	C	***I***	***E***	***I***	S	***S***	***E***	***S***	Q	V	I	***I***
106/1^72R,76I^	CQ then QN	72R,76I-PfCRT^K1^	1.13 ± 0.07	0.76 ± 0.07	0.87 ± 0.06	***R***	***I***	***E***	***I***	S	***S***	***E***	***S***	Q	V	I	***I***
106/1^76I,352K^	CQ then QN	76I,352K-PfCRT^K1^	0.91 ± 0.09	0.93 ± 0.25	1.28 ± 0.07	C	***I***	***E***	***I***	S	***S***	***E***	***S***	***K***	V	I	***I***
106/1^76I,352R^	CQ then QN	76I,352R-PfCRT^K1^	1.58 ± 0.02	0.99 ± 0.16	0.88 ± 0.02	C	***I***	***E***	***I***	S	***S***	***E***	***S***	***R***	V	I	***I***
106/1^76I,369F^	CQ then AMT^d^	76I,369F-PfCRT^K1^	3.53	0.28	0.98	C	***I***	***E***	***I***	S	***S***	***E***	***S***	Q	***F***	I	***I***
K1AM	CQ then AMT	163R,356V-PfCRT^K1^	0.95	0.72	n.d.	C	***I***	***E***	***T***	***R***	***S***	***E***	***S***	Q	V	***V***	***I***

^a^The 106/1-derived lines were generated by Cooper and colleagues [11, 12, 14] and the K1AM line was produced by Johnson et al. [15].

^b^The susceptibilities of the parasite lines to CQ, quinine (QN), and quinidine (QD) were collated from Cooper et al. [11, 12, 14] and Johnson et al. [15]. The drug resistance index is the IC_50_ for a drug in a given strain or line divided by the IC_50_ determined in the same study in a drug-sensitive reference strain (i.e., 106/1, HON, or GC03). The indices were calculated from a single study or are the mean of values from two (± range/2) or ≥ three (± SEM) studies.

^c^Residues that differ from the wild-type amino acid sequence (e.g., the PfCRT sequence carried by 3D7, D10, and HON parasites) are shown in bold italics.

^d^CQ-resistant parasites are hypersensitive to amantadine [13, 15].

We compared the abilities of these PfCRT^K1^ variants to transport CQ, quinine, quinidine, and amantadine when expressed in *Xenopus* oocytes and complemented the amantadine work with assays that detected the PfCRT-mediated transport of the drug in the parasite. Our findings indicate that the hypersensitivity of the 106/1^76I^ line to quinine arises from the drug exerting two antiplasmodial effects—its normal anti-hemozoin activity as well as potent inhibition of PfCRT^CQR^, which likely kills the parasite by blocking the normal function of the transporter. By contrast, the hypersensitivity of CQ-resistant parasites to amantadine is due to its PfCRT^CQR^-mediated efflux from the DV into the cytosol, where it appears to gain better access to its antiplasmodial target. In both cases, the mutations that suppress hypersensitivity cause a substantial or complete reduction in the capacity of PfCRT^CQR^ for CQ transport, thus explaining why rescue from hypersensitivity reinstates the parasite’s sensitivity to CQ.

## Results

### Expression of PfCRT^K1^ isoforms at the oocyte plasma membrane

**Fig 1:**
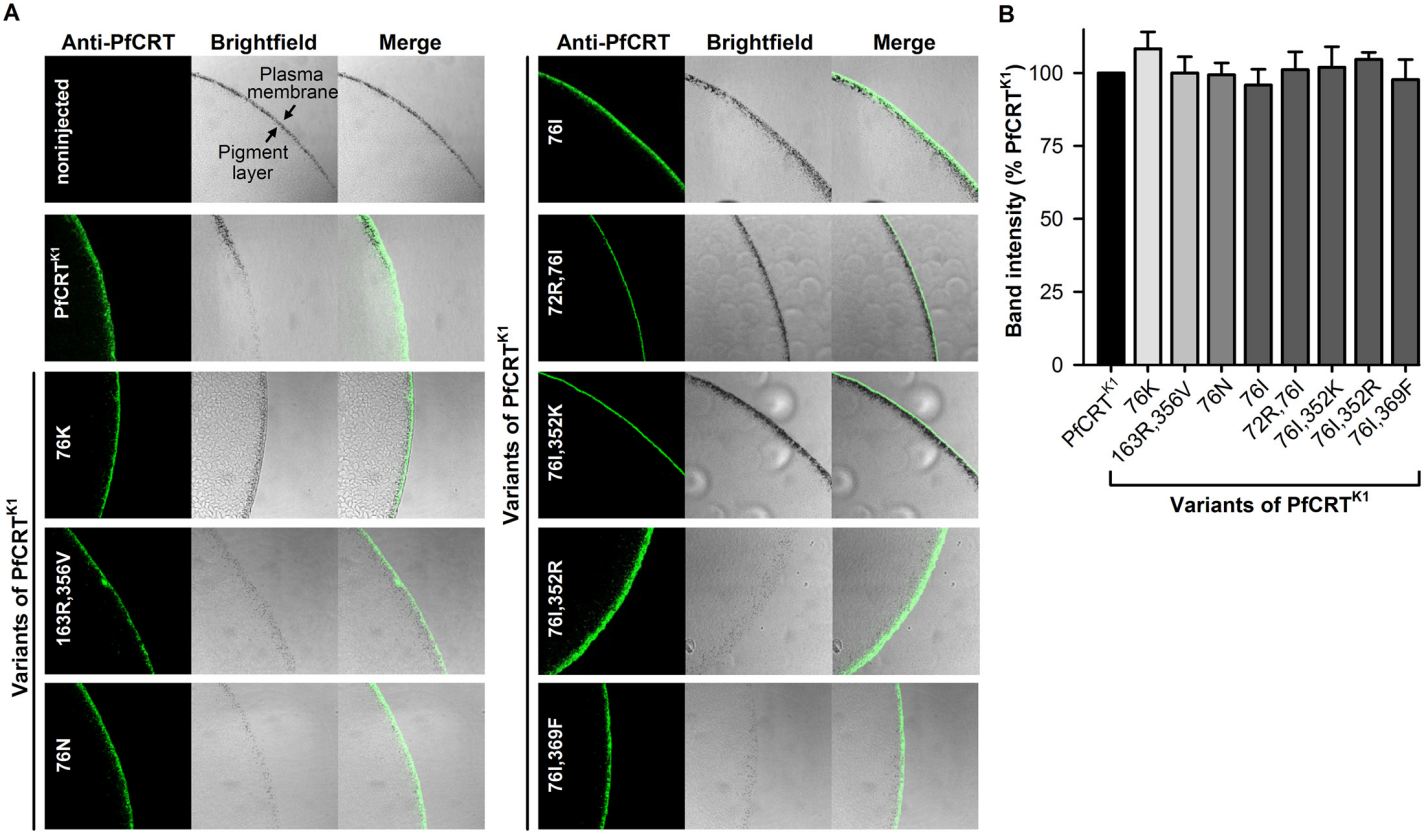
Isoforms of PfCRT^K1^ localize to the surface of *Xenopus* oocytes. (A) Immunofluorescence microscopy was used to localize PfCRT in the oocyte. In each case, the expression of the PfCRT variant resulted in a fluorescent band external to the pigment layer, indicating that the protein was expressed in the oocyte plasma membrane. The band was not present in noninjected oocytes. The images are representative of at least two independent experiments (performed using oocytes from different frogs), within which images were obtained from a minimum of three oocytes per oocyte type. (B) The level of PfCRT protein in the oocyte membrane was semiquantified using a western blot method [42]. The analysis included PfCRT^K1^ as a positive control, to which the other band intensity values were normalized. The data are the mean + SEM of at least five independent experiments (performed using oocytes from different frogs), within which measurements were averaged from two independent replicates. There were no significant differences in expression levels between constructs (*P* > 0.05; one-way ANOVA); hence, all of the PfCRT variants were present at similar levels in the oocyte membrane.

We used a previously-described version of the PfCRT^K1^ coding sequence [42] to generate the isoforms of *pfcrt* carried by the 106/1 and K1 parasite lines (Table 1). This version of the *pfcrt* sequence has been codon-harmonized for expression in *Xenopus* oocytes and encodes a retention motif-free form of PfCRT that expresses in a functional form at the oocyte plasma membrane [42]. We conducted immunofluorescence assays (Fig 1A) to confirm the localization of each of the PfCRT^K1^ variants to the oocyte plasma membrane and used a semiquantitative western blot analysis [42] to establish that they were present at comparable levels in the oocyte membrane (Fig 1B). Hence, any differences in drug transport activity between these isoforms of PfCRT can be attributed to differences in their transport properties rather than differences in expression.

### Quinoline transport via PfCRT largely correlates with quinoline resistance

**Fig 2:**
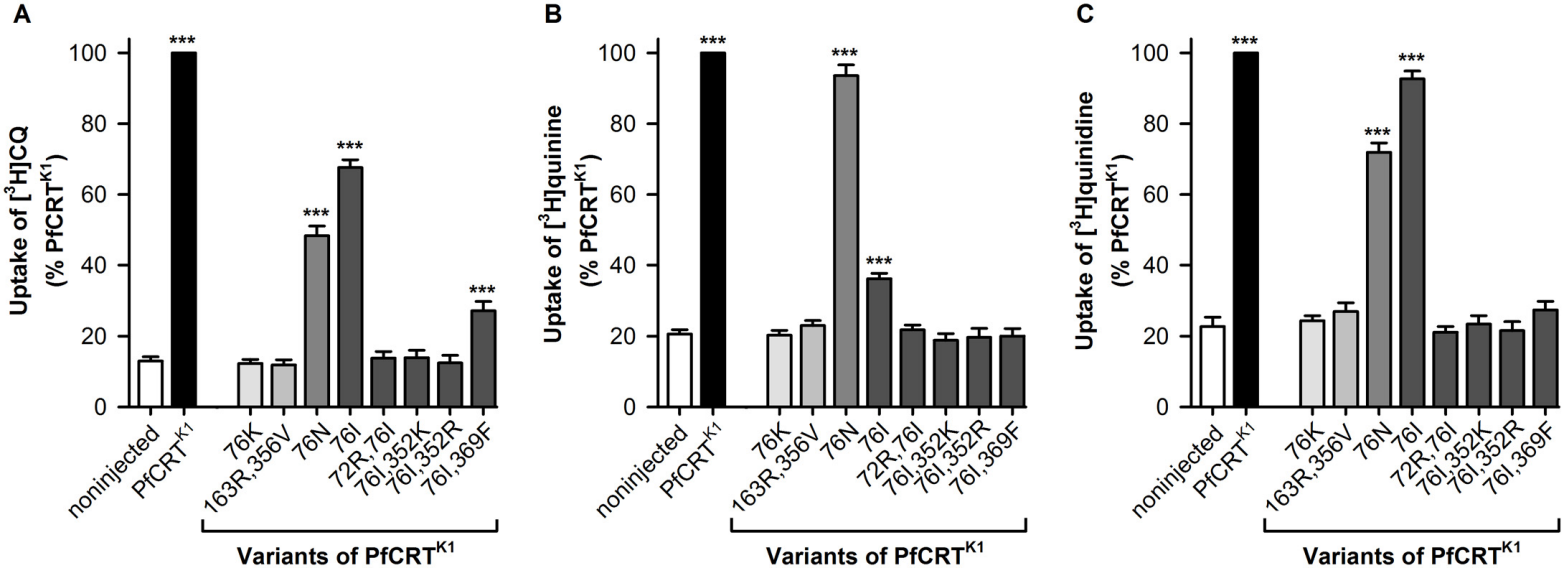
CQ, quinine, and quinidine transport activities differ significantly between isoforms of PfCRT^K1^. Noninjected oocytes accumulate low levels of CQ, quinine, and quinidine via simple diffusion of the neutral species of the drug [29, 30]. This represents the background level of drug accumulation. In all cases, the concentration of the [^3^H]drug under study was 0.25 μM. (A) The uptake of [^3^H]CQ was measured at pH 6.0 and in the presence of 15 μM unlabeled CQ. The rates of CQ uptake (pmol per oocyte/h) in noninjected oocytes and oocytes expressing PfCRT^K1^ were 0.98 ± 0.13 and 6.75 ± 0.94, respectively. (B, C) The uptake of [^3^H]quinine or [^3^H]quinidine was measured at pH 5.0 and in the presence of 1 μM of the respective unlabeled drug. The rates of uptake (pmol per oocyte/h) in noninjected oocytes and oocytes expressing PfCRT^K1^ were 0.10 ± 0.01 and 0.55 ± 0.03, respectively, for quinine and 0.063 ± 0.010 and 0.38 ± 0.08, respectively, for quinidine. Note that the relatively low total concentration of quinine and quinidine (1.25 μM) used in these assays enabled the detection of [^3^H]quinine transport via the 76I-PfCRT^K1^ isoform. However, this low drug concentration also resulted in rates of quinine and quinidine transport that were 10–15 times lower than those measured for CQ (a difference which corresponds well with the ~12-fold reduction in the total concentration of quinine or quinidine relative to the total concentration of CQ). In all panels, drug uptake is expressed relative to that measured in oocytes expressing PfCRT^K1^ and the data are the mean + SEM of at least five independent experiments (performed using oocytes from different frogs), within which measurements were made from 10 oocytes per treatment. The asterisks denote a significant difference in drug uptake between the noninjected treatment and that measured in oocytes expressing a variant of PfCRT: *P < 0.05; **P < 0.01; ***P < 0.001 (one-way ANOVA).

To understand the mechanisms underlying the inverse drug susceptibilities of the 106/1- and K1-derived lines, we characterized the transport properties of the respective PfCRT^K1^ variants in *Xenopus* oocytes. A key advantage of the oocyte system is that it allows the transport activity of PfCRT to be studied directly and in isolation, without confounding effects such as the binding of drugs to heme or interactions of the compound with other parasite targets or transporters. The direction of [^3^H]drug transport in this system is from the acidic extracellular medium (pH 5.0–6.0) into the oocyte cytosol (pH 7.1–7.2 [30]), which corresponds to the efflux of protonated drug from the acidic DV (pH 5.0–5.5 [43, 44]) into the parasite cytosol (pH 7.3 [45]). Noninjected oocytes take up [^3^H]CQ, [^3^H]quinine, and [^3^H]quinidine to low levels via simple diffusion of the neutral species of the drug (Fig 2A–2C); this represents the background level of [^3^H]drug accumulation [29, 30]. We found that none of the PfCRT^K1^ isoforms carried by CQ-sensitive exhibited CQ transport activity when expressed in oocytes (Fig 2A). By contrast, the expression of PfCRT^K1^ variants from CQ-resistant parasites caused significant increases in the accumulation of CQ. Indeed, we observed a positive correlation between the ability of a PfCRT^K1^ variant to transport CQ and the magnitude of *in vitro* CQ resistance exhibited by the respective parasite line (R^2^ value of 0.856; S2A Fig). We conclude that the mutations which suppress the quinine-hypersensitivity of the 106/1^76I^ line (C72R, Q352K, or Q352R) simultaneously re-sensitize the parasite to CQ by abrogating the protein’s capacity to efflux CQ from the DV. Likewise, a mutation that suppresses the hypersensitivity of 106/1^76I^ parasites to amantadine (V369F), also caused a marked reduction in the ability of 76I-PfCRT^K1^ to transport CQ, which explains why the 106/1^76I,369F^ line displayed a relatively low level of resistance to CQ.

Similar results were obtained when we examined the uptake of [^3^H]quinine and [^3^H]quinidine (Fig 2B and 2C and S2 Fig). That is, the capacities of the different PfCRT^K1^ isoforms to transport quinine or quinidine generally correlated with the *in vitro* responses of the parasite lines to these drugs. Key exceptions included the quinine hypersensitivities of the 106/1^76I^ and 106/1^76I,369F^ lines, which were not readily reconciled with the low quinine transport activity of 76I-PfCRT^K1^ and the lack of quinine transport via 76I,369F-PfCRT^K1^, respectively. The modest capacity of 76I-PfCRT^K1^ for quinine transport was nonetheless abolished by the introduction of C72R, Q352K, or Q352R. Another exception was the moderate ability of 76N-PfCRT^K1^ to transport quinidine, which was at odds with the sensitivity of 106/1^76N^ parasites to this drug.

Together, these findings revealed a strong relationship between the magnitude of CQ, quinine, or quinidine resistance exhibited by a given 106/1 or K1 parasite and the capacity of its PfCRT protein for mediating the efflux of the respective drug from the DV. However, the mechanism underpinning the quinine-hypersensitivity caused by the introduction of T76I into PfCRT^K1^, and why the subsequent addition of C72R, Q352K, or Q352R negates this response, remained unclear.

### High-affinity binding of quinine to 76I-PfCRT^K1^ causes hypersensitivity to quinine

We undertook experiments to determine whether the kinetics of 76I-PfCRT^K1^- and 76N-PfCRT^K1^-mediated transport could explain why these proteins cause unexpected responses to quinine and quinidine, respectively. All of the PfCRT^K1^ isoforms we had identified as possessing significant CQ, quinine, or quinidine transport activity (Fig 2A–2C) were included for study. The resulting Michaelis-Menten plots (S3 Fig) yielded a Michaelis–Menten constant (K_m_) and maximum velocity (V_max_) for each drug and PfCRT isoform (Table 2). Across all three drugs, the K_m_ values increased in the order 76I-PfCRT^K1^ < PfCRT^K1^ < 76N-PfCRT^K1^, whereas the V_max_ values increased in the order 76I-PfCRT^K1^ < 76N-PfCRT^K1^ ≤ PfCRT^K1^. Thus, we conclude that T76I increases the affinity of PfCRT^K1^ for its quinoline substrates, but that this change is accompanied by a significant decrease in its maximum rate of transport. By contrast, T76N consistently decreased the affinity of PfCRT^K1^ for its quinoline substrates, and either had little effect on the V_max_ (quinine) or caused a marked reduction in the V_max_ (CQ and quinidine). Interestingly, the introduction of V369F into 76I-PfCRT^K1^ significantly reduced the protein’s affinity for CQ (from 247 to 415 μM) and further reduced its maximum rate for CQ transport (from 29.3 to 13.8 pmol per oocyte/h), while also abrogating its ability to transport quinine and quinidine (Fig 2B and 2C). Our finding that 76I,369F PfCRT^K1^ is a very low-affinity and low-capacity transporter of CQ is again consistent with the modest level of CQ resistance exhibited by the 106/1^76I,369F^ parasite line.

**Table 2:** Kinetic parameters for the transport of CQ, quinine, and quinidine via variants of PfCRT^K1^.

PfCRT variant	Apparent K_m_ (μM)^a^ ^,^ ^b^			Apparent V_max_ (pmol per oocyte/h)		
	CQ	Quinine	Quinidine	CQ	Quinine	Quinidine
PfCRT^K1^	282 ± 5 *(9)*	28.8 ± 2.7 *(7)*	84 ± 1 *(6)*	72 ± 3 *(9)*	10.3 ± 0.8 *(7)*	24.4 ± 1.6 *(7)*
76N-PfCRT^K1^	303 ± 5^e,f^ *(7)*	41.0 ± 2.6^f^ *(6)*	94 ± 4^c,f^ *(6)*	48.0 ± 4.5^c,f^ *(7)*	13.0 ± 1.0^d,f^ *(6)*	14.1 ± 0.9^e,g^ *(6)*
76I-PfCRT^K1^	247 ± 7^c,f^ *(5)*	0.42 ± 0.04^c,f^ *(4)*	24.1 ± 3.0^f^ *(6)*	29.3 ± 7.2^c,f^ *(5)*	0.09 ± 0.01^c,f^ *(4)*	7.2 ± 0.5^e,g^ *(6)*
76I,369F-PfCRT^K1^	415 ± 25^c^ *(5)*	n.d.	n.d.	13.8 ± 1.3^c^ *(5)*	n.d.	n.d.

^a^PfCRT-mediated transport was calculated by subtracting the uptake of drug measured in the control oocytes (oocytes expressing 76K-PfCRT^K1^) from that in oocytes expressing PfCRT^K1^, 76N-PfCRT^K1^, 76I-PfCRT^K1^, or 76I,369F-PfCRT^K1^. The data are shown in S3 Fig.

^b^All values are the mean ± SEM of multiple independent experiments (performed using oocytes from different frogs), within which measurements were made from 10 oocytes per treatment. The exact *n* values are indicated in parentheses.

The *P* values determined from an ANOVA were less than ^c^0.001, ^d^0.01, or ^e^0.05 for comparisons with PfCRT^K1^ within the same drug treatment and kinetic parameter.

The *P* values determined from an ANOVA were less than ^f^0.001 or ^g^0.01 for comparisons between 76N-PfCRT^K1^ and 76I-PfCRT^K1^ within the same drug treatment and kinetic parameter.

Remarkably, we found that 76I-PfCRT^K1^ possesses an extraordinarily high affinity for quinine and an extraordinarily low V_max_ for quinine transport; its K_m_ was ~70 times lower, and its V_max_ ~115 times lower, than the value obtained for PfCRT^K1^. This combination of kinetic properties indicates that 76I-PfCRT^K1^ binds extremely tightly to quinine and only occasionally translocates the drug. Thus, quinine will clog the binding site of 76I-PfCRT^K1^, which should greatly diminish the ability of the protein to transport its natural substrate. These findings suggest that quinine-hypersensitivity results from quinine exerting two killing effects in 106/1^76I^ parasites—the inhibition of hemozoin formation and the inhibition of PfCRT’s normal physiological role (which is essential for parasite survival [39, 40]). The low level of quinine transport mediated by 76I-PfCRT^K1^ was not detected following the introduction of C72R, Q352K, Q352R, or V369F (Fig 2B). Of these mutations, Q352R and Q352K each fully return 106/1^76I^ parasites to quinine-sensitive status, C72R causes a substantial but incomplete rescue from quinine-hypersensitivity, and V369F confers a modest but significant suppression of the parasite’s hypersensitivity to quinine (Table 1). Taken together, our data indicate that the insertion of a positively-charged residue into the substrate-binding cavity of 76I-PfCRT^K1^ greatly diminishes or abolishes its interaction with quinine (and its ability to transport CQ and quinidine), thereby reversing the parasite’s hypersensitivity to quinine (and simultaneously re-sensitizing it to CQ and quinidine). Our finding that these effects are achieved to a much lesser degree by V369F, which instead introduces a bulky hydrophobic residue into 76I-PfCRT^K1^, indicates that electrostatic repulsion is the key mechanism underpinning the dramatic decrease in the protein’s ability to bind and translocate protonated quinolines.

Our kinetic analyses also revealed that 76N-PfCRT^K1^ is unique in being both a low-affinity and a low-capacity transporter of quinidine. By comparison, PfCRT^K1^ mediated the low-affinity but high-capacity transport of quinidine, and 76I-PfCRT^K1^ was a high-affinity, low-capacity transporter of quinidine. Thus, the sensitivity of 106/1^76N^ parasites to quinidine is likely due to the inability of 76N-PfCRT^K1^ to decrease the concentration of quinidine within the DV to sub-toxic levels.

### I356T increases quinine transport via 76I-PfCRT^K1^

We recently reported [42] that the CQ transport activity of PfCRT^K1^ is ~20% higher than that of PfCRT^Dd2^, even though the two proteins differ only at position 356 (Table 1). However, the I356T mutation also demonstrated epistasis. That is, depending on the nature and number of the mutations already present, its introduction increased, decreased, or had no effect on the ability of PfCRT to transport CQ [42]. We therefore investigated whether the introduction of I356T decreases the ability of PfCRT^K1^ variants to transport quinine and quinidine. We measured the uptake of [^3^H]quinine or [^3^H]quinidine in oocytes expressing either a PfCRT^K1^ variant (PfCRT^K1^, 76N-PfCRT^K1^, or 76I-PfCRT^K1^) or its I356T counterpart (PfCRT^Dd2^, 76N-PfCRT^Dd2^, or 76I-PfCRT^Dd2^). Consistent with our previous observation with CQ transport, we found that the rate of quinine and quinidine uptake mediated by PfCRT^K1^ was 17–35% greater than that measured for PfCRT^Dd2^ (S4 Fig). Moreover, we observed the same relationship, only considerably more exaggerated, between the 76N variants of PfCRT^K1^ and PfCRT^Dd2^ and, in the case of quinidine transport, between 76I-PfCRT^K1^ and 76I-PfCRT^Dd2^. The single exception to this pattern was quinine transport via 76I-PfCRT^K1^, which showed a 30–40% decrease relative to its PfCRT^Dd2^ counterpart. Our findings establish a key role for position 356 in the attainment of a high level of quinoline transport activity, but also confirm the epistatic nature of this position.

### Isoforms of PfCRT from amantadine-hypersensitive parasites transport amantadine

**Fig 3:**
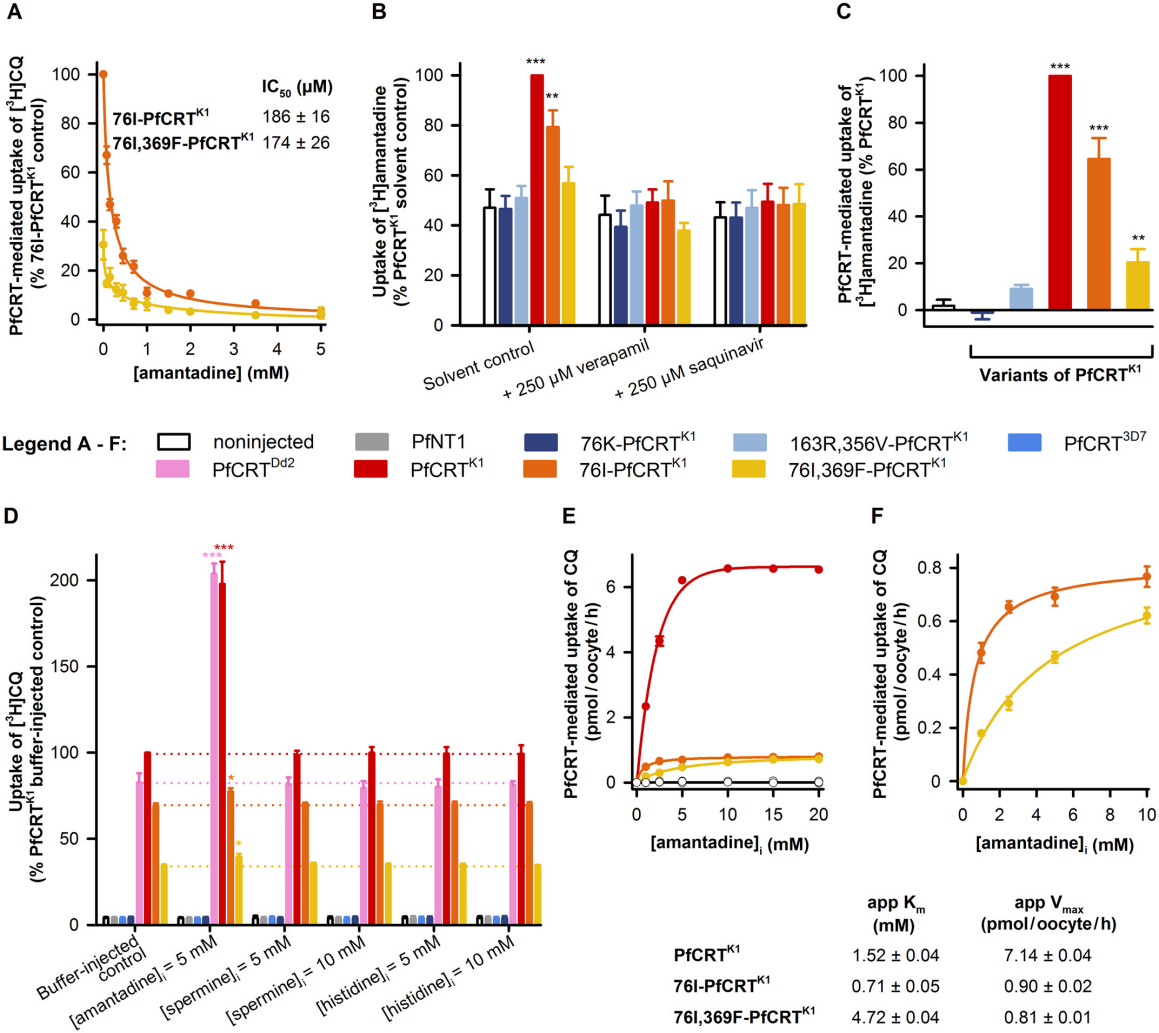
Isoforms of PfCRT^K1^ from amantadine-hypersensitive parasites transport amantadine. (A) Amantadine inhibits the transport of [^3^H]CQ via 76I-PfCRT^K1^ and 76I,369F-PfCRT^K1^ in a concentration-dependent manner (IC_50_s of 186 ± 16 and 174 ± 26 μM, respectively). (B) The uptake of [^3^H]amantadine (0.146 μM) in the absence and presence of verapamil or saquinavir. The measurements were undertaken at pH 5.0 and in the presence of 50 μM unlabeled amantadine. (C) The PfCRT-mediated transport of [^3^H]amantadine. Using the data shown for the solvent control in panel B, the component of [^3^H]amantadine transport attributable to PfCRT was calculated by subtracting the background level of accumulation (i.e., the average of the uptake measured in noninjected oocytes and oocytes expressing 76K-PfCRT^K1^) from that measured for each of the oocyte types. The rates of amantadine uptake (nmol per oocyte/h) in noninjected oocytes and PfCRT^K1^-expressing oocytes were 5.4 ± 0.9 and 12 ± 2.8, respectively. The asterisks denote a significant difference from the noninjected control: *P < 0.05; **P < 0.01; ***P < 0.001 (one-way ANOVA). (D) *Trans*-stimulation of PfCRT-mediated [^3^H]CQ transport by unlabeled amantadine. The oocytes were microinjected with a buffer control or with buffer containing amantadine, spermine, or histidine. The estimated intracellular concentrations ([compound]_i_) are indicated. The asterisks denote a significant difference from the relevant buffer-injected control. (E) Concentration-dependence of the *trans*-stimulation of [^3^H]CQ uptake by amantadine. The oocytes were microinjected with buffer containing amantadine to achieve an estimated [amantadine]_i_ of 1 to 20 mM. The app K_m_ and app V_max_ values are the apparent kinetic parameters for the *trans*-stimulatory effect of amantadine. The data show CQ uptake above that measured in the relevant buffer-injected control; the total rates of CQ uptake are presented in S6 Fig. The noninjected data overlays the data obtained with oocytes expressing PfNT1, PfCRT^3D7^, or 76K-PfCRT^K1^. (F) A magnified plot of the 76I-PfCRT^K1^ and 76I,369F-PfCRT^K1^ data from panel E. In all panels, the data are the mean ± SEM of five independent experiments (performed using oocytes from different frogs), within which measurements were made from 10 oocytes per treatment. Where not shown, error bars fall within the symbols.

We next sought to understand why CQ-resistant parasites are hypersensitive to amantadine and how certain mutations in PfCRT (e.g., T76K, S163R, or V369F) countercheck this response. Given that we had already ascertained that the PfCRT^K1^ variants carrying T76K or S163R do not possess significant quinoline transport activity (Fig 2A–2C), we focused our initial investigations on 76I,369F-PfCRT^K1^, as it mediates a detectable level of CQ transport. To test the possibility that hypersensitivity to amantadine is due to it clogging the substrate-binding cavity of PfCRT, and that this effect is alleviated by the addition of V369F, we compared the ability of unlabeled amantadine to inhibit the transport of [^3^H]CQ via 76I-PfCRT^K1^ and 76I,369F-PfCRT^K1^ (Fig 3A). Somewhat surprisingly, there was little difference in the resulting half-maximum inhibitory concentrations (IC_50_s; listed in Fig 3A). Moreover, these IC_50_s were much higher than those obtained in the oocyte system for established inhibitors of PfCRT^CQR^, such as the quinine dimer Q_2_C (1.4 ± 0.2 μM [46]), saquinavir (13 ± 1 μM [47]), and verapamil (30 ± 3 μM [30]). Since amantadine is a relatively low-affinity inhibitor of both 76I-PfCRT^K1^ and 76I,369F-PfCRT^K1^, it is unlikely that the hypersensitivity of CQ-resistant parasites to amantadine is due to it exerting an anti-PfCRT^CQR^ effect.

This result led us to examine whether amantadine hypersensitivity is instead a consequence of significant changes in the ability of PfCRT to transport amantadine. We found that oocytes expressing PfCRT^K1^ or 76I-PfCRT^K1^ showed a marked increase in [^3^H]amantadine accumulation relative to noninjected oocytes (Fig 3B and 3C). By contrast, oocytes expressing 76K-PfCRT^K1^ or 163R,356V-PfCRT^K1^ failed to take up [^3^H]amantadine above the background level of accumulation, and 76I,369F-PfCRT^K1^ displayed only a very low (but statistically significant) level of amantadine transport activity. Moreover, [^3^H]amantadine transport via PfCRT^K1^, 76I-PfCRT^K1^, and 76I,369F-PfCRT^K1^ was inhibited by the PfCRT^CQR^ inhibitors verapamil and saquinavir. These findings suggest that hypersensitivity to amantadine arises from the ability of PfCRT^CQR^ isoforms to transport amantadine, and that mutations such as T76K, S163R, and V369F reverse this response by substantially decreasing or abolishing amantadine transport activity.

The signal-to-background ratio we obtained for [^3^H]amantadine transport in the oocyte system was around 2 (Fig 3B), which is relatively modest when compared with that obtained for quinine and quinidine transport (which typically produce ratios of 4–7; e.g., Fig 2B and 2C and S4 Fig) or CQ transport (which typically produces a ratio of 8–25; e.g., Figs 2A and 3D and S5 Fig). We therefore interrogated the amantadine transport properties of PfCRT further by conducting a series of *trans*-stimulation experiments. Many transporters reorientate more quickly from one face of the membrane to the other when a substrate is bound compared with when the transporter is empty. Transporters that display this characteristic can be *trans*-stimulated; in the case of PfCRT^CQR^, the uptake of [3H]CQ from the external solution will be accelerated by the presence of an unlabeled substrate on the cytosolic face of the membrane. We therefore measured the uptake of [^3^H]CQ into oocytes which had been microinjected with a buffer control or with buffer containing unlabeled amantadine, spermine, or histidine. Spermine (a polycation) and histidine (a weak base) do not appear to interact with PfCRT^Dd2^ (S5 Fig and Martin et al. [30]) and were therefore included as extra negative controls. In addition, oocytes expressing an unrelated *P*. *falciparum* transporter (the nucleoside transporter PfNT1 [48]) were included as a further negative control. We found that amantadine did not affect the accumulation of [^3^H]CQ in noninjected oocytes or oocytes expressing 76K-PfCRT^K1^, PfCRT^3D7^, or PfNT1 (Fig 3D). Moreover, neither spermine nor histidine altered [^3^H]CQ uptake in any of the oocyte types. By contrast, amantadine *trans*-stimulated the transport of [^3^H]CQ into oocytes expressing PfCRT^K1^, PfCRT^Dd2^, 76I-PfCRT^K1^, or 76I,369F-PfCRT^K1^, albeit to different extents; the respective increases in the rate of CQ influx (in pmol per oocyte/h) were 6.4 ± 0.2, 6.7 ± 0.3, 0.68 ± 0.04, and 0.43 ± 0.02. We extended this analysis by measuring the concentration-dependence of the *trans*-stimulation of PfCRT^K1^, 76I-PfCRT^K1^, and 76I,369F-PfCRT^K1^ (Fig 3E and 3F and S6 Fig). A least-squares fit of the Michaelis-Menten equation to the data yielded apparent kinetic parameters for the *trans*-stimulatory effect of amantadine (listed in Fig 3). These results revealed that the addition of V369F to 76I-PfCRT^K1^ causes a 6.2- to 7.2-fold increase in the concentration of amantadine required for the half-maximal *trans*-stimulation of the transporter.

Taken together, our work shows that PfCRT variants from parasites that are hypersensitive to amantadine possess the ability to transport this drug, and that the introduction of mutations that suppress amantadine hypersensitivity either abolish (T76K and S163R) or substantially decrease (V369F) the protein’s capacity for amantadine transport. These findings led us to propose that amantadine, which should sequester within the DV via weak-base trapping, exerts its main antiplasmodial effect outside of the DV and that the PfCRT-mediated leak of the drug back into the cytosol results in amantadine hypersensitivity. We therefore utilized a set of parasite assays to test this hypothesis *in situ*.

### PfCRT effluxes amantadine from the DV of amantadine-hypersensitive parasites

**Fig 4:**
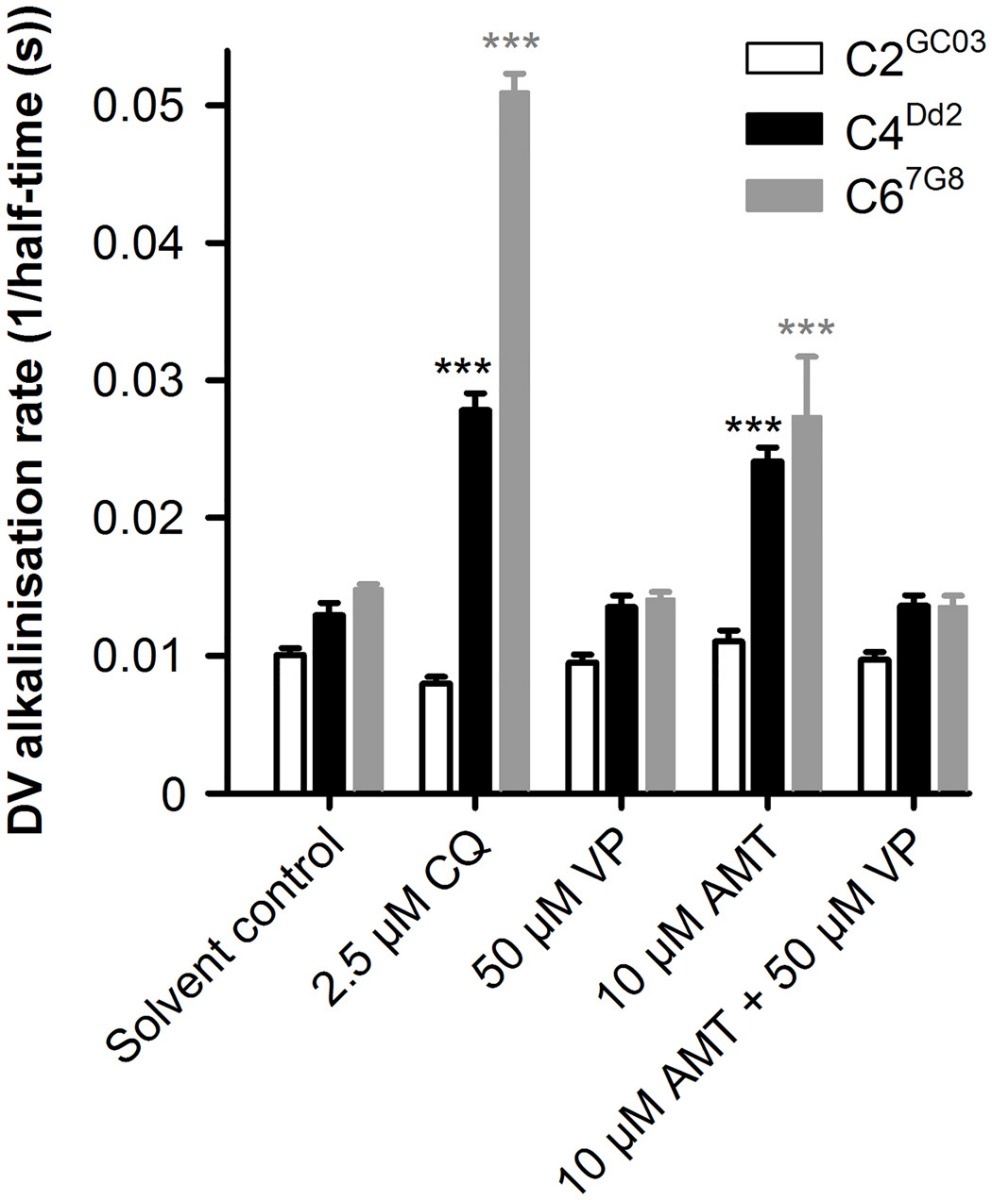
Amantadine is a substrate of PfCRT^Dd2^ and PfCRT^7G8^, and not of wild-type PfCRT, *in situ*. The rate of concanamycin A-induced DV alkalinization (expressed as the inverse of the half-time for DV alkalinization) in the C2^GC03^ (CQ-sensitive, expressing PfCRT^3D7^), C4^Dd2^ (CQ-resistant, expressing PfCRT^Dd2^), and C6^7G8^ (CQ-resistant, expressing PfCRT^7G8^) transfectant lines was measured in the absence and presence of amantadine (AMT), CQ, or verapamil (VP). The effect of amantadine on the DV alkalinization rate was also measured in the presence of verapamil (final concentration of 50 μM). The drugs were added to suspensions of saponin-isolated trophozoite-stage parasites containing fluorescein-dextran in their DVs 4 min before the addition of concanamycin A (100 nM). CQ was added at a concentration of 2.5 μM and the concentration of amantadine was 10 μM. Consistent with previous studies, the addition of CQ elevated the rate of alkalinization in both of the CQ-resistant lines and had a small buffering effect in the C2^GC03^ parasites [34–36, 46]. The biological basis for the difference in the rate of CQ-dependent alkalinization between the two CQ-resistant lines is currently unclear, but could be due to differences in (1) the expression of PfCRT, (2) the transport properties of the two PfCRT isoforms, (3) the volume of the DV, and/or (4) the concentration of PfCRT’s natural substrates within the DV. The data are the mean + SEM of five independent experiments (performed on different days). The asterisks denote a significant difference from the relevant solvent control: ***P < 0.001 (one-way ANOVA).

The function of PfCRT can be investigated in its native environment by employing an assay that indirectly detects the movement of protonated drugs out of the DV [34–36]. This method uses a fluorescent pH-sensitive probe to measure an outward leak of protons, which manifests as an increase in the rate of alkalinization of the DV. A drug-induced proton leak arises when a weak-base drug enters the acidic DV in its unprotonated form and is effluxed in its protonated form. We applied this assay to a set of *P*. *falciparum* transfectants (the C2^GC03^, C4^Dd2^, and C6^7G8^ lines [2]) that are isogenic except for their *pfcrt* allele, which encodes either PfCRT^3D7^ (C2^GC03^) or a PfCRT^CQR^ isoform of the protein (PfCRT^Dd2^ in C4^Dd2^ and PfCRT^7G8^ in C6^7G8^). CQ was included as a positive control and consistent with previous observations [34–36, 46], it increased the rate of DV alkalinization in the CQ-resistant C4^Dd2^ and C6^7G8^ lines and was without effect in the CQ-sensitive C2^GC03^ line (Fig 4). We obtained similar results with amantadine and also showed that verapamil inhibits the amantadine-associated leak of protons from the DV of C4^Dd2^ and C6^7G8^ parasites. These findings confirm that amantadine accumulates within the DV via weak-base trapping and also provide *in situ* evidence of the ability of PfCRT^CQR^ isoforms to transport amantadine back into the cytosol.

If amantadine hypersensitivity results from the PfCRT-mediated efflux of the drug from the DV, we would expect that inhibitors of PfCRT^CQR^ would reduce this response. We tested this hypothesis by determining the susceptibility of the isogenic lines to amantadine in the absence and presence of verapamil or chlorpheniramine [49] (another established inhibitor of PfCRT^CQR^). These experiments, which included CQ as a control as well as CQ-resistant (Dd2) and CQ-sensitive (3D7) reference strains, entailed using a fluorescence-based method to measure parasite growth in the presence of increasing concentrations of amantadine or CQ. The resulting IC_50_s (Table 3) provided two salient findings. First, the data verified the striking hypersensitivity of CQ-resistant parasites to amantadine. Secondly, in all cases this hypersensitivity was partially suppressed by the PfCRT inhibitors. Thus, we conclude that the hypersensitivity of the 106/1^76I^ line, and of other CQ-resistant parasites, to amantadine arises from the PfCRT^CQR^-mediated redistribution of the drug from the DV into the cytosol, where it gains better access to its main antiplasmodial target.

**Table 3:** *In vitro* antiplasmodial activities of CQ and amantadine against CQ-sensitive and CQ-resistant *P*. *falciparum* parasites.

Strain/line^b^	IC_50_ ^a^					
	CQ (nM)^c^			Amantadine (μM)^c^		
	Control	+ 1 μM VP	+ 1 μM CP	Control	+ 1 μM VP	+ 1 μM CP
3D7	25 ± 1.1	24 ± 0.9	22 ± 0.7	344 ± 8.9	352 ± 9.1	351 ± 11
Dd2	151 ± 4.8^d^	25 ± 1.0^g^	11 ± 0.04^g^	11 ± 0.1^d^	55 ± 0.6^f^	64 ± 0.9^g^
C2^GC03^	27 ± 1.6	26 ± 1.3	22 ± 3.1	335 ± 9.5	341 ± 11	348 ± 13
C4^Dd2^	149 ± 4.6^d^	26 ± 0.09^g^	18 ± 0.01^g^	8.4 ± 0.06^d^	57 ± 0.6^g^	66 ± 1.0^g^
C6^7G8^	87 ± 2.1^d^	25 ± 0.2^g^	20 ± 0.06^g^	43 ± 2.9^d^	78 ± 4.3^e^	88 ± 5.4^f^

^a^The IC_50_ values are the mean ± SEM of five independent experiments (performed on different days), within which measurements were averaged from 3 replicates.

^b^Field-derived strains: 3D7 (CQ-sensitive; Africa); Dd2 (CQ-resistant; Indochina/Laos). Isogenic *pfcrt* transfectant lines: C2^GCO3^ (CQ-sensitive); C4^Dd2^ (CQ-resistant); C6^7G8^ (CQ-resistant).

^c^Measurements were made in the absence and presence of verapamil (VP) or chlorpheniramine (CP).

^d^The *P* values determined from a one-way ANOVA were less than 0.001 for comparisons with the relevant CQ-sensitive strain or line (and within either the CQ or amantadine treatment).

The *P* values determined from a one-way ANOVA were less than ^e^0.001, ^f^0.01, or ^g^0.05 for comparisons between the control treatment and the relevant VP or CP treatment from the same strain or line.

## Discussion

**Fig 5:**
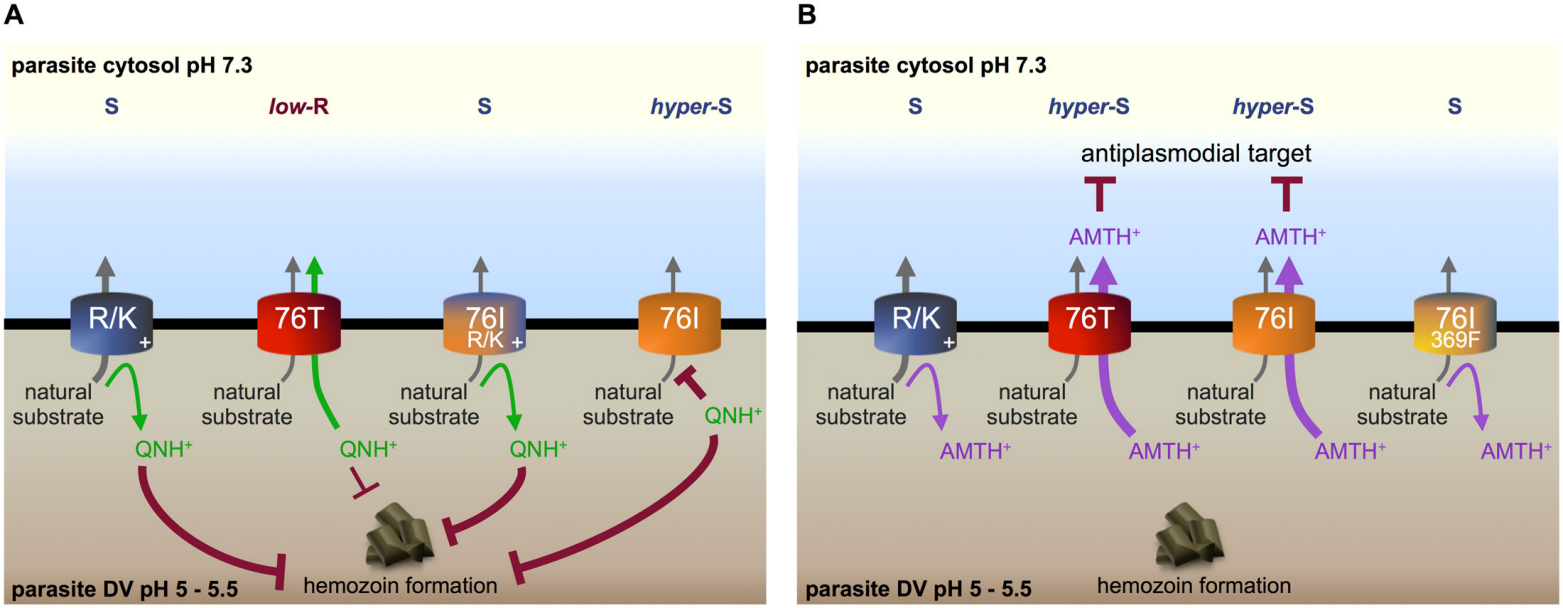
Molecular mechanisms for the drug hypersensitivities induced by PfCRT isoforms in the malaria parasite. (A) The variants of PfCRT^K1^ that contain 72R, 76K, 163R, 352K, or 352R (R/K) do not possess significant quinine (QN) transport activity. The drug would therefore remain in the parasite’s DV where it exerts an anti-hemozoin effect that kills the parasite, which is consistent with the QN-sensitive (S) status of the respective lines. PfCRT^K1^ (76T) is able to transport QN out of the DV and thereby imparts low-level resistance (*low*-R) to QN. By contrast, 76I-PfCRT^K1^ has an extremely high affinity for QN coupled with an extremely low maximum rate of transport. This causes QN to clog the binding site of 76I-PfCRT^K1^, thereby blocking the transport of the natural substrate. Hence, the QN-hypersensitivity (*hyper*-S) observed in 106/1^76I^ parasites results from QN exerting two killing effects—anti-hemozoin and anti-PfCRT^CQR^. The gain of a positively-charged residue at position 72 or 352 (76I R/K) prevents the interaction of the transporter with QN and returns the parasites to QN-sensitive status. (B) Amantadine (AMT) is a relatively poor inhibitor of both 76I-PfCRT^K1^ and 76I,369F-PfCRT^K1^, making it unlikely that the AMT-hypersensitivity of CQ-resistant parasites is due to an anti-PfCRT^CQR^ effect. The isoforms of PfCRT from AMT-hypersensitive parasites (PfCRT^K1^ and 76I-PfCRT^K1^) have the ability to transport this weak-base drug out of the DV (where it accumulates) whereas those from AMT-sensitive parasites either do not possess significant AMT transport activity (e.g., 76K-PfCRT^K1^ and 163R,356V-PfCRT^K1^; R/K) or transport AMT with low affinity and low capacity (76I,369F-PfCRT^K1^). The data therefore converge on a scenario in which AMT exerts its main antimalarial activity in the cytosol and AMT-hypersensitivity arises from the redistribution of the drug from the DV into the cytosol via a PfCRT^CQR^ variant (e.g., PfCRT^K1^ or 76I-PfCRT^K1^).

Our work provides mechanistic explanations for the patterns of inverse susceptibility induced by PfCRT. First, we confirmed our previous observation [42] of there being a positive correlation between the capacity of a given PfCRT isoform for mediating CQ transport and the magnitude of CQ resistance achieved by the respective parasite (S7A Fig) and extended this relationship to include, with the notable exception of the QN-hypersensitive lines, a positive correlation between the quinine or quinidine transport activity of PfCRT and the parasite’s *in vitro* responses to these drugs (Fig 5A and S7B Fig). Moreover, we showed that in most cases, the isoforms of PfCRT^K1^ that possessed CQ transport activity also transported quinine and quinidine and *vice versa*. Together, these findings confirm a common role for PfCRT in reducing the accumulation of CQ, quinine, and quinidine within the DV and explain the tendency of CQ-resistant parasites to exhibit decreased susceptibilities to quinine and quinidine. However, our results resolve this phenomenon further by providing a fundamental insight into PfCRT-induced drug phenotypes: whether a given isoform of PfCRT alters the parasite’s susceptibility to a drug, and to what extent and in which direction, depends on the kinetics of the drug’s transport via PfCRT.

A key example of this principle is the quinine-hypersensitivity conferred by 76I-PfCRT^K1^. Our work reveals that the introduction of 76I into PfCRT^K1^ has the remarkable effect of transforming the protein into an exceedingly high-affinity, low-capacity transporter of quinine. These highly abnormal kinetic properties will cause the drug to clog PfCRT’s substrate-binding cavity, which should block the transport of the natural substrate and thereby prevent the protein from fulfilling its essential physiological role (Fig 5A). Thus, we propose that hypersensitivity to quinine results from the drug exerting at least two killing effects in 106/1^76I^ parasites—anti-hemozoin and anti-PfCRT^CQR^. This hypothesis provides mechanistic explanations for two previously abstruse observations: (1) despite dramatic differences in their quinine responses, 106/1^76I^ and 106/1 parasites accumulate similar levels of quinine [11] and (2) quinine and CQ produce a synergistic interaction in 106/1^76I^ parasites, whereas a slightly antagonistic interaction occurs in the 106/1^76T^ line [12]. In the case of the first observation, the lack of a difference in quinine accumulation concurs with the exceedingly low capacity of 76I-PfCRT^K1^ for transporting quinine. In regard to the second phenomenon, the synergistic interaction in 106/1^76I^ is readily reconcilable with the ability of quinine to adhere to the binding cavity of 76I-PfCRT^K1^, as this should both inhibit the normal function of the protein and block the efflux of CQ from the DV. The slightly antagonistic effect observed in the 106/1^76T^ parasites is likely to result from the two drugs competing to bind to heme (CQ is the more potent inhibitor of hemozoin formation [25, 27]) and/or other differences in their activities (e.g., CQ also appears to inhibit the glutathione-dependent degradation of heme, whereas quinine is thought to lack this activity [50]).

Cooper and colleagues reported a third perplexing characteristic of the 106/1^76I^ line that may likewise arise from quinine’s ability to block the normal function of 76I-PfCRT^K1^. The presence of verapamil typically re-sensitizes CQ-resistant parasites to CQ (and to quinine and quinidine) and this resistance-reversing effect was evident for all three drugs in the 106/1^76T^ and 106/1^76N^ lines [11, 12]. It was also apparent in the 106/1^76I^ parasites, but only in the CQ and quinidine treatments. In the quinine treatment, verapamil exerted the opposite effect; i.e., it *decreased* the sensitivity of the 106/1^76I^ line to quinine and thus partially suppressed the parasite’s hypersensitivity to this drug [11, 12]. One possible explanation for this highly unusual observation is a scenario in which verapamil competes with quinine for binding to 76I-PfCRT^K1^, and that the natural substrate is better able to access the translocation pore when verapamil is bound relative to when quinine is bound. Given that the substrate-binding site of PfCRT^Dd2^ has been shown to behave as a large polyspecific cavity that can bind at least two molecules simultaneously [29], it is conceivable that verapamil and quinine interact with the binding cavity differently and that verapamil presents somewhat less of an obstacle to the transport of the natural substrate via 76I-PfCRT^K1^ than does quinine. Such a scenario would provide a mechanistic explanation for the verapamil-induced reduction in quinine’s activity against the 106/1^76I^ line.

We recently reported that CQ-resistant parasites are hypersensitive to dimers of quinine, and that this appears to be due to these molecules inhibiting both heme detoxification and PfCRT^CQR^ function (the quinine dimers are potent inhibitors of PfCRT^Dd2^ but are not translocated by the transporter) [46]. Hence, the anti-PfCRT^CQR^ activity of quinine in the 106/1^76I^ parasites is not simply an intriguing but extraneous biological oddity; it signifies that one of the parasite’s key modulators of drug resistance is itself a druggable antimalarial target.

Our findings suggest that less dramatic changes in the kinetics of transport via PfCRT can also significantly affect the parasite’s response to a drug. 76N-PfCRT^K1^ decreases the parasite’s susceptibility to CQ and quinine while being without effect on its response to quinidine [11, 12, 14]. A simple interpretation of these observations would be that 76N-PfCRT^K1^ transports CQ and quinine but fails to recognize quinidine. However, we found that 76N-PfCRT^K1^ maintains the ability to transport quinidine, but that its affinity for the drug is slightly reduced, and that its maximum rate of quinidine transport is significantly reduced, relative to the kinetics of quinidine transport via PfCRT^K1^. The low-capacity, low-affinity nature of quinidine transport via 76N-PfCRT^K1^ will limit the protein’s ability to reduce the accumulation of the drug within the DV and hence could explain why 76N-PfCRT^K1^ has little net effect on the parasite’s susceptibility to quinidine (S7B Fig).

The hypersensitivity of CQ-resistant parasites to amantadine was first reported several decades ago [13], but the molecular basis for this phenomenon has remained unknown. We demonstrate that amantadine hypersensitivity cannot be explained by an anti-PfCRT^CQR^ effect and is instead attributable to a second mechanism. Our work shows that amantadine sequesters within the acidic environment of the DV and that the hypersensitivity of CQ-resistant parasites to amantadine results from the ability of PfCRT^CQR^ isoforms to efflux the drug from the DV into the parasite cytosol (Fig 5B). These findings indicate that amantadine acts on a target outside of the DV and that it exerts its antiplasmodial activity to greater effect when proteins such as PfCRT^Dd2^, PfCRT^7G8^, PfCRT^K1^, and 76I-PfCRT^K1^ leak the accumulated drug back into the cytosol. The antiviral target of amantadine is a proton channel [51] and it is possible that the drug likewise targets an essential cation channel or transporter in the parasite.

All of the mutations that introduced a positively-charged residue into PfCRT^K1^ abolished its ability to transport CQ, quinine, quinidine, and amantadine. Hence, in addition to confirming the importance of electrostatic repulsion in preventing interactions between PfCRT and protonated drugs, our results suggest that the transporter’s ability to transport protonated quinolines out of the DV (and thereby confer quinoline resistance) is fundamentally connected to its ability to transport protonated amantadine out of the DV (and thereby induce amantadine hypersensitivity). Furthermore, the detection in field isolates of T76K, C72R, or S163R in otherwise PfCRT^CQR^ isoforms [5] indicates that these types of changes could be occurring in the parasite population in response to selection forces exerted by other drugs and/or because, in the absence of CQ, these parasites are fitter than their CQ-resistant counterparts (most PfCRT^CQR^ isoforms impart a fitness cost [52–55]). Such a phenomenon appears to have taken place in French Guiana; Pelleau and colleagues [56] recently showed that another mutation that introduces a positive charge into PfCRT^CQR^ (C350R) emerged in a CQ-resistant population following the withdrawal of CQ and is attributed with reinstating these parasites to CQ-sensitive status.

Our findings also provide a foundation for understanding and exploiting clinically-relevant cases of reciprocal collateral sensitivity. Most of the current treatments for uncomplicated malaria are combination therapies that pair an artemisinin derivative (all of which are metabolised into dihydroartemisinin) with a quinoline-related partner drug—of which the most widely used is lumefantrine [57]. Cases of severe malaria, as well as *Plasmodium*-infected pregnant women, are typically treated with combinations that include an antibiotic, such as clindamycin. It is, therefore, worth noting that several *in vitro* studies have observed isoforms of PfCRT^CQR^ to induce hypersensitivity to lumefantrine (with 1.5–3.2-fold decreases in the IC_50_ [16, 17, 56, 58–61]), artemisinin and/or dihydroartemisinin (with 1.9–3.7-fold decreases in the IC_50_ [6, 11, 17, 58, 59, 62]) as well as to a number of antibiotics [10, 63–67]—including clindamycin [10, 63, 65, 68]. Moreover, multiple clinical trials undertaken in malarious regions throughout the world have associated the wild-type protein (i.e., PfCRT^3D7^) with significant reductions in the parasite’s susceptibility to artemether-lumefantrine [16, 69–71], such that (1) the administration of artemether-lumefantrine was found to cause significant selection of CQ-sensitive parasites carrying PfCRT^3D7^ and (2) the presence of parasites carrying PfCRT^3D7^ prior to artemether-lumefantrine treatment was associated with an increased risk of recrudescence. It is possible that the hypersensitivity of CQ-resistant parasites to lumefantrine, the artemisinins, and/or to the antibiotics results from one of the mechanisms we describe in this study. For instance, the antibiotics are either known or expected to act on targets outside of the DV [63, 72, 73], but due to their weak-base nature, these drugs will sequester within the acidic environment of this organelle. Hence, it is plausible that the antibiotics gain greater access to their antimalarial target by being transported into the cytosol via PfCRT^CQR^. For these antibiotics as well as for lumefantrine and the artemisinins, and for the many other pharmacons that display enhanced activity against CQ-resistant parasites, the set of assays outlined in our study offer the means to determine which of the two mechanisms are involved, or whether an altogether different mechanism is responsible. Such insights have the potential to contribute to the formulation of rational approaches for maintaining and extending the useful lifespan of many antimalarials by exploiting the opposing selection forces they exert upon PfCRT.

## Materials and Methods

### Generation of PfCRT coding sequences and synthesis of cRNA

The coding sequences of the different isoforms of PfCRT were generated via site-directed mutagenesis using the primer pairs listed in S1 Table and an approach described previously [42]. The mutations were introduced into a codon-harmonized version of the PfCRT sequence that had been inserted into the pGEM-He-Juel oocyte expression vector [74]. This sequence encodes a version of PfCRT that is free of endosomal-lysosomal trafficking motifs and which is therefore expressed at the plasma membrane of *Xenopus laevis* oocytes [30, 42]. All of the resulting coding sequences were verified by sequencing (undertaken by the ACRF Biomolecular Resource Facility, ANU). The plasmids were linearized with SalI (ThermoFisher Scientific) and 5’-capped complementary RNA (cRNA) was synthesized using the mMessage mMachine T7 transcription kit (Ambion), and then purified with the MEGAclear kit (Ambion).

### Harvest, preparation, and microinjection of *X*. *laevis* oocytes

Ethical approval of the work performed with the *X*. *laevis* frogs was obtained from the Australian National University Animal Experimentation Ethics Committee (Animal Ethics Protocol Number A2013/13) in accordance with the Australian Code of Practice for the Care and Use of Animals for Scientific Purposes. Oocytes were harvested and prepared as described in full elsewhere [9]. Briefly, sections of ovary were harvested from adult female frogs (purchased from NASCO) via a minor surgical procedure and single, de-folliculated oocytes were prepared using collagenase D (Roche). Stage V-VI oocytes were microinjected with cRNA (20 ng per oocyte) encoding PfCRT or PfNT1 and were stored at 16–18°C in OR^2+^ buffer (82.5 mM NaCl, 2.5 mM KCl, 1 mM MgCl_2_, 1 mM Na_2_HPO_4_, 5 mM HEPES, 1 mM CaCl_2_, and 50 μg/mL gentamycin; pH 7.8).

### Oocyte membrane preparation and western blot analysis

The preparation of oocyte membranes and the semi-quantification of PfCRT protein was carried out using a protocol described in detail elsewhere [42]. Protein samples prepared from oocyte membranes were separated on a 4–12% Bis-Tris SDS-polyacrylamide gel (Life Technologies) and transferred to a Protran 0.45 μM nitrocellulose membrane (Amersham, GE Healthcare Life Sciences). The membranes were probed with rabbit anti-PfCRT antibody (concentration of 1:4,000; Genscript) followed by horseradish peroxidase-conjugated goat anti-rabbit antibody (1:8,000; Life Technologies, cat. no. 656120). Validation of the specificity of the anti-PfCRT antibody has been published in detail elsewhere [42]. The PfCRT band for each variant was detected by chemiluminescence (Pierce), quantified using the Image J software [75], and expressed as a percentage of the intensity measured for the PfCRT^K1^ band. Total protein staining was used to evaluate sample loading and efficiency of transfer as outlined previously [42]. Between five and seven independent experiments were performed (on oocytes from different frogs), and in each experiment measurements were averaged from two independent replicates.

### Immunofluorescence of oocytes expressing PfCRT

Immunofluorescence analyses were performed on oocytes three days post-injection using a method adapted from Weise et al. [76]. Unless specified otherwise, both the incubation and wash steps were conducted at room temperature with gentle shaking or rotation. The volume of the incubation solution was 500 μL and the washes were performed with 1 mL of the specified solution. Six oocytes from each treatment type were fixed for 30 min in a solution of phosphate-buffered saline (PBS) and 4% v/v paraformaldehyde and then washed (10 min) three times in PBS. The oocytes were permeabilized with 100% methanol for 20 min (-20°C, without shaking) and washed (10 min) three times in PBS. A blocking solution (4% w/v bovine serum albumin (BSA), 2% v/v normal goat serum (Life Technologies), and 0.1% v/v Triton X-100 in PBS) was applied for 2 h, after which the oocytes were incubated overnight at 4°C in a second blocking solution (4% w/v BSA and 2% v/v normal goat serum in PBS). The samples were then incubated for a further 4 h at room temperature before the blocking solution was replaced with a solution containing the rabbit anti-PfCRT antibody (1:100 in 1.5% w/v BSA and 0.01% v/v Triton X-100 in PBS) and the samples were incubated for 4 h at room temperature and then overnight at 4°C. Three 10-min washes were performed in PBS supplemented with 1.5% w/v BSA and all of the remaining steps were undertaken in the dark (and at room temperature). The Alexa Fluor 488 donkey anti-rabbit antibody (1:500; Molecular Probes, cat. no. A-21206) was incubated with the samples for 4 h in PBS supplemented with 4% w/v BSA and 2% v/v normal goat serum, after which three 10-min washes were performed in PBS.

The oocytes were post-fixed with paraformaldehyde (3.7% v/v in PBS) for 30 min, washed twice (15 min) in 2 mL of PBS, and then dehydrated with a series of incubations in solutions of increasing ethanol content. The solutions (in order of administration) were: 30% v/v ethanol in PBS, 50% v/v ethanol in PBS, 70% v/v ethanol in ultrapure water, 90% v/v ethanol in ultrapure water, and 100% ethanol. In each case, the samples were briefly washed in the ethanol-containing solution before a 15-min incubation was performed. A further two incubations in 100% ethanol were conducted before the oocytes were embedded in an acrylic resin using the Technovit 7100 plastic embedding system (Kulzer). Briefly, the samples were incubated with 500 μL embedding solution (50% v/v Technovit 7100 in 100% ethanol) for 2 h, after which the oocytes were incubated overnight in 500 μL of a second embedding solution (1% w/v Technovit 7100 ‘hardener 1’ in Technovit 7100). A further two incubations (≥2 h each) were performed in the second embedding solution before all of the solution was removed and 800 μL of a third embedding solution (6.66% v/v Technovit 7100 ‘hardener 2’ and 1% w/v Technovit 7100 ‘hardener 1’ in Technovit 7100) was added. After the samples had set (≥4 d), a microtome was used to obtain ~4 μm slices, which were dried on microscope slides. Coverslips with a drop of ProLong Gold Antifade Mountant (Life Technologies) were placed over the slices and sealed with nail polish.

Images of the slices were obtained with a Leica Sp5 inverted confocal laser microscope (Leica Microsystems) using the 63x objective. Excitation was achieved with a 488 nm argon laser and the emissions were captured using a 500–550 nm filter. Images were acquired using the Leica Application Suite Advanced Fluorescence software (Leica Microsystems). At least two independent experiments were performed (on oocytes from different frogs) for each oocyte type, within which slices were examined from a minimum of three oocytes. All of the slices taken from oocytes expressing a PfCRT variant displayed a fluorescent band above the pigment layer (i.e., consistent with the localization of PfCRT to the plasma membrane) that was not present in noninjected oocytes.

### Drug transport assays

The radiolabeled drugs were purchased from American Radiolabeled Chemicals ([^3^H]CQ, [^3^H]quinine, and [^3^H]quinidine) or Moravek ([^3^H]amantadine). The uptake into oocytes of [^3^H]CQ (0.25 μM; 20 Ci/mmol), [^3^H]quinine (0.25 μM; 20 Ci/mmol), [^3^H]quinidine (0.25 μM; 20 Ci/mmol), or [^3^H]amantadine (0.146 μM; 137 mCi/mmol) was measured 3–6 days post-injection. Unless specified otherwise, the drug influx assays were conducted over 1.5–2 h at 27.5°C and in the presence of a low concentration of the unlabeled drug (CQ, 15 μM; quinine and quinidine, 1 μM; amantadine, 50 μM). The reaction buffer was either ND96 pH 5.0 ([^3^H]quinine, [^3^H]quinidine, and [^3^H]amantadine transport assays) or ND96 pH 6.0 ([^3^H]CQ transport assays) and contained 96 mM NaCl, 2 mM KCl, 1 mM MgCl_2_, and 1.8 mM CaCl_2_ supplemented with either 20 mM homo-PIPES (pH 5.0 buffer) or 10 mM MES and 10 mM Tris-base (pH 6.0 buffer). For each treatment, 10 oocytes were transferred to a 5 mL polystyrene round bottom tube (Falcon) and washed twice with 3.5 mL of ND96 buffer, with the residual buffer removed by pipette. Influx commenced with the addition of 100 μL of ND96 buffer supplemented with the radiolabeled and unlabeled drug and, where specified, an unlabeled inhibitor (e.g., verapamil, saquinavir, or CQ). The assay was terminated by removing the reaction buffer with a pipette and washing the oocytes twice with 3.5 mL of ice-cold ND96 buffer. Each oocyte was transferred to a separate well of a white 96-well plate (NUNC or PerkinElmer), incubated overnight at room temperature in 30 μL of 10% SDS, and mixed on an orbital shaker the following day for approximately 5 min. The lysed oocyte was then combined with 150 μL of MicroScint-40 microscintillant (PerkinElmer), the plate covered with a TopSeal-A (PerkinElmer), and the radioactivity measured with a PerkinElmer MicroBeta^2^ microplate liquid scintillation analyzer.

Note that noninjected oocytes and oocytes expressing PfCRT^3D7^ take up [^3^H]CQ to similar (low) levels via simple diffusion of the neutral species of the drug; this represents the ‘background’ level of [^3^H]CQ accumulation in oocytes (refer to Martin et al. [30] for full data and discussion). There is also no detectable difference in the accumulation of [^3^H]quinine between noninjected and PfCRT^3D7^-expressing oocytes, nor does the accumulation of [^3^H]quinidine differ between these two oocyte types, and this background level of uptake has likewise been attributed to simple diffusion [29, 30].

The kinetic parameters for CQ, quinine, and quinidine transport via different isoforms of PfCRT (Table 1 and S3 Fig) were determined in SigmaPlot Windows Version 11.0 (Systat Software Inc.) by a least-squares fit of the Michaelis-Menten equation (v = V_max_ substrate]/(K_m_ + [substrate]) to the data.

The IC_50_ values presented in Fig 3A were determined in SigmaPlot by a least-squares fit of the equation *y* = *y*_min_ + [(*y*_max_—*y*_min_)/(1 + ([inhibitor]/IC_50_)*c*] to the data, where *y* is PfCRT-mediated CQ transport, *y*_min_ and *y*_max_ are the minimum and maximum values of *y*, and *c* is a fitted constant. PfCRT-mediated CQ transport was calculated by subtracting the uptake measured in the control oocytes (oocytes expressing 76K PfCRT^K1^) from that in oocytes expressing 76I-PfCRT^K1^ or 76I,369F-PfCRT^K1^.

A subset of experiments measured the ability of unlabeled amantadine to *trans*-stimulate the uptake of [^3^H]CQ into the oocyte. Immediately prior to the commencement of the experiment, the oocytes (days 3–4 post-injection of the cRNA) were microinjected with either amantadine (estimated intracellular concentrations of 1, 2.5, 5, 10, 15, or 20 mM) or a control treatment (buffer, spermine, or histidine; the estimated intracellular concentrations of spermine and histidine were 5 and 10 mM). The volume injected was 50 nL and the intracellular concentrations were calculated using previous estimates of the volume of stage V-VI oocytes (~400 nL [77]). The resealed oocytes were then incubated at 16–18°C in OR^2+^ buffer for approximately 5 min and the influx of [^3^H]CQ was measured as described above. For each amantadine concentration, the rate of CQ influx above that measured in the relevant buffer-injected control was calculated and a least-squares fit of the Hill equation (*y* = V_max_[amantadine]_i_^n^/(K_m_^n^ + [amantadine]_i_^n^) to the data was performed in SigmaPlot, where *y* is the reaction velocity and *n* is the Hill coefficient. This analysis yielded Hill coefficients of 1.63 ± 0.12, 1.49 ± 0.07, and 1.46 ± 0.05 for PfCRT^K1^, 76I-PfCRT^K1^ and 76I,369F-PfCRT^K1^, respectively. These values indicated that CQ and amantadine either bind independently of one another, or are slightly cooperative (i.e., the binding of one drug enhances the affinity of the transporter for the second drug). The Michaelis-Menten equation was then fitted to the data to derive the kinetic parameters for the *trans*-stimulation of [^3^H]CQ transport by amantadine.

In most cases, at least five independent experiments were performed (on different days and using oocytes from different frogs), and within each experiment measurements were made from 10 oocytes per treatment.

### Culture of *P*. *falciparum*-infected erythrocytes

The use of human blood in this study was approved by the Australian National University’s Human Research Ethics Committee. The CQ-sensitive strain ‘3D7’ (isolated from the Netherlands but probably of African origin [78]), the CQ-resistant strain ‘Dd2’ (isolated from Southeast Asia), and three *pfcrt* transfectant lines [2] (C2^GC03^, C4^Dd2^, and C6^7G8^) were cultured and synchronized as described previously [79, 80]. In the C4^Dd2^ and C6^7G8^ lines, the wild-type *pfcrt* allele of the CQ-sensitive ‘GC03’ strain has been replaced with the *pfcrt* allele from Dd2 or from the CQ-resistant ‘7G8’ strain (isolated from Brazil), respectively. C2^GC03^ is a CQ-sensitive recombinant control that retains the wild-type *pfcrt* allele (i.e., PfCRT^3D7^). C6^7G8^ contains an additional mutation (I351M) in PfCRT that is not present in 7G8 parasites [36]. The parasite lines were maintained in the presence of the selection agents blasticidin (5 μM; Sigma-Aldrich) and WR99210 (5 nM; Jacobus Pharmaceuticals). These selection agents were not present during the experiments.

### *P*. *falciparum* H^+^ efflux assay

Saponin-isolated trophozoite-stage parasites containing the membrane-impermeant pH-sensitive fluorescent indicator fluorescein-dextran (10,000 MW; Life Technologies) in their DVs were prepared as outlined elsewhere [36]. The isolated parasites were washed and suspended in a saline solution (125 mM NaCl, 5 mM KCl, 1 mM MgCl_2_, 20 mM glucose, 25 mM HEPES; pH 7.1) at a density of 1–3 × 10^7^ cells/mL. The fluorometry experiments were performed as described previously [35]. The pH of the DV was monitored at 37°C using a PerkinElmer Life Sciences LS50B fluorometer with a dual excitation Fast Filter accessory (excitation 490 and 450 nm; emission 520 nm). The experiments entailed monitoring the alkalinization of the DV upon addition of the V-type H^+^-ATPase inhibitor concanamycin A (100 nM; Sigma-Aldrich), in the presence or absence of the drugs of interest. Half-times for DV alkalinization (t1/2) were calculated as outlined elsewhere [34]. In all cases, five independent experiments were performed on different days.

### *P*. *falciparum* drug susceptibility

Parasite proliferation was measured in 96-well plates using a fluorescent DNA-intercalating dye [81] and a protocol described in detail previously [82]. Briefly, cell suspensions containing erythrocytes infected with ring-stage parasites (hematocrit and parasitemia of approximately 2% and 1%, respectively) were incubated at 37°C for 72 h. The samples were frozen, thawed, and then processed by the addition of 100 μL (per well) of SYBR Safe DNA Gel Stain (Molecular Probes; 0.2 μL/mL) in a lysis buffer (20 mM Tris, 5 mM EDTA, 0.008% w/v saponin, and 0.08% v/v Triton X-100; pH 7.5). The fluorescence emanating from each well was measured immediately using a Tecan Infinite M1000 PRO microplate reader (excitation 490 nm; emission 520 nm) and the average fluorescence from wells containing the highest concentration of the drug was subtracted from the resulting values. The level of parasite proliferation in the presence of each drug concentration was expressed as a percentage of the proliferation measured in the absence of the drug for which the IC_50_ was being determined. The IC_50_s were determined in SigmaPlot by a least-squares fit of the equation *y* = *a*/ [1 ([drug]/IC_50_)*c*] to the data, where *y* is the percent parasite proliferation, *a* is the maximum change in the percent parasite proliferation, and *c* is a fitted constant. In all cases, five independent experiments were performed (on different days), and within each experiment measurements were averaged from three replicates.

### Statistics

All errors cited in the text and shown in the figures represent the SEM. Statistical comparisons were made using one-way ANOVAs in conjunction with Tukey’s multiple comparisons test.

## Supporting Information

S1 Fig Predicted topology of PfCRT showing the mutations present in the PfCRT^K1^ variants characterized in this study. PfCRT is predicted to contain 10 α-helical transmembrane domains (TMDs) and to be orientated in the digestive vacuole (DV) membrane with the N- and C-termini extending into the parasite cytosol [83]. The positions of the mutated residues in PfCRT^K1^ are indicated with black circles. The key CQ resistance-associated mutation (K76T) is represented as a red square. The purple circles show the locations of the additional residues that are mutated in the variants of PfCRT^K1^. The box attached to each polymorphic residue lists the (non-wild-type) amino acid(s) that occur at that position. The predicted roles of the TMDs are as follows: 4 and 9 (outlined in dark green) are implicated in the binding and translocation of substrates, TMDs 3 and 8 (boxed in light green) are thought to assist in the binding and translocation of the substrate and may also influence the substrate-specificity of the transporter, TMDs 1, 2, 6, and 7 (boxed in black) may be involved in recognizing and discriminating between substrates, and TMDs 5 and 10 (outlined in mid-green) are thought to play a role in the formation of homo-dimers [83].
(TIFF)

S2 Fig Plots of the quinoline transport activities of PfCRT versus the parasite *in vitro* drug responses. (A-C) The CQ, quinine, or quinidine transport activity of a given PfCRT^K1^ isoform (calculated from the data presented in Fig 2) was plotted against the *in vitro* resistance index for the relevant drug and parasite line (listed in Table 1). Where not shown, error bars fall within the symbols. There was a positive correlation between the *in vitro* response of a parasite line to CQ and the CQ transport properties of the corresponding isoform of PfCRT^K1^ (panel A: R^2^ = 0.856). This is consistent with our previous observation of a positive correlation (R^2^ = 0.858; [42]) in an analysis performed with seven field isoforms of PfCRT, but contrasts with the work of Roepe and colleagues, who have not detected a relationship between PfCRT^CQR^-mediated CQ transport and the parasite’s response to CQ [84, 85]. In panel B, the quinine transport activities of many of the PfCRT^K1^ isoforms correlated positively with the *in vitro* responses of the parasites to this drug. However, two key exceptions included the data for the highly QN-hypersensitive lines 106/1^76I^ and 106/1^76I,369F^. Likewise, in panel C, a positive correlation between the capacity of a given PfCRT^K1^ isoform to transport quinidine and the relevant parasite’s *in vitro* quinidine resistance index held for all of the parasites bar the QD-sensitive line 106/1^76N^. Refer to the main text for a discussion of these outlying points. Note also that other genetic elements, such as the amplification or mutation of the parasite’s multidrug resistance protein 1 (PfMDR1) and the altered expression of other genes, can contribute to the quinoline resistance phenotype [7, 86–90]. Hence, the CQ resistance index obtained for the 106/1^76I^ line, which is higher than what might be expected from the CQ transport activity of 76I-PfCRT^K1^, may be due to changes in the expression of one or more genes, including *pfmdr1* [14, 89]. High-level CQ resistance has been associated with reductions in the *pfmdr1* copy number and a corresponding decrease in the expression of *pfmdr1* [91, 92]. In this regard it is worth noting that the 106/1^76I^ line contains a single copy of *pfmdr1*, whereas the 106/1, 106/1^76T^, and 106/1^76N^ lines possess two copies, and this difference has been confirmed to result in a lower level of *pfmdr1* expression in the 106/1^76I^ parasites [14, 89]. Hence, it is likely that the relatively high level of CQ resistance displayed by 106/1^76I^ is the product of the effects of the CQ transport activity of 76I-PfCRT^K1^, the reduced expression of PfMDR1, and perhaps changes that have occurred elsewhere in the parasite’s transcriptome. Thus, while this study and our previous work [42] indicate that the quinoline transport activities of a number of PfCRT variants correlate positively with the *in vitro* responses of the respective parasites to these drugs, changes elsewhere in the genome and transcriptome can modulate the level of quinoline resistance conferred by PfCRT^CQR^.
(TIF)

S3 Fig Isoforms of PfCRT^K1^ exhibit different CQ, quinine, and quinidine transport kinetics when expressed in oocytes. (A) The uptake of [^3^H]CQ (0.25 μM) was measured at pH 6.0 and over an extracellular concentration range of 10 to 1250 μM unlabeled CQ. (B) The uptake of [^3^H]quinine (0.25 μM) was measured at pH 5.0 and the extracellular concentration of unlabeled quinine ranged between 1 and 300 μM (PfCRT^K1^ and 76N-PfCRT^K1^) or 0.25 and 12 μM (76I-PfCRT^K1^). The inset shows a magnified plot of the 76I-PfCRT^K1^ data. (C) The uptake of [^3^H]quinidine (0.25 μM) was measured at pH 5.0 and the extracellular concentration of unlabeled quinidine ranged between 1 and 500 μM (PfCRT^K1^ and 76I-PfCRT^K1^) or 1 and 750 μM (76N-PfCRT^K1^). In all cases, the rate of PfCRT-mediated drug uptake was calculated by subtracting the rate measured in oocytes expressing 76K-PfCRT^K1^ from that measured in oocytes expressing PfCRT^K1^, 76N-PfCRT^K1^, 76I-PfCRT^K1^, or 76I,369F-PfCRT^K1^ at each drug concentration. The Michaelis-Menten equation was fitted to the resulting data using nonlinear regression. The rates of drug uptake are the mean ± SEM of multiple independent experiments (performed using oocytes from different frogs), within which measurements were made from 10 oocytes per treatment. Where not shown, error bars fall within the symbols. The exact *n* values, as well as the kinetic parameters derived from these data, are presented in Table 2.
(TIF)

S4 Fig Quinine and quinidine transport activities of the 76I and 76N isoforms of PfCRT^Dd2^ and PfCRT^K1^. Noninjected oocytes accumulate low levels of quinine and quinidine via simple diffusion of the neutral species of the drug [29]. This represents the background level of drug accumulation. The uptake of (A) [^3^H]quinine and (B) [^3^H]quinidine was measured at pH 5.0 and in the presence of 1 μM of the respective unlabeled drug. The concentration of the [^3^H]drug was 0.25 μM. The rates of uptake (pmol per oocyte/h) in noninjected oocytes and oocytes expressing PfCRT^Dd2^ were 0.10 ± 0.01 and 0.44 ± 0.04, respectively, for quinine and 0.05 ± 0.01 and 0.30 ± 0.07, respectively, for quinidine. In both panels, drug uptake is expressed relative to that measured in oocytes expressing PfCRT^Dd2^. The rates of quinine and quinidine uptake mediated by PfCRT^K1^ were 1.2–1.3 times that measured for PfCRT^Dd2^, whereas the 76N-PfCRT^K1^ protein possessed 3.1 times the quinine transport activity, and 3.2 times the quinidine transport activity, of 76N-PfCRT^Dd2^. Moreover, the rate of quinidine transport mediated by 76I-PfCRT^K1^ was 1.5 times that measured for 76I-PfCRT^Dd2^. The single exception to this trend was the rate of quinine transport via 76I-PfCRT^K1^, which was 0.55 times that measured for its PfCRT^Dd2^ counterpart. The data are the mean + SEM of at least five independent experiments (performed using oocytes from different frogs), within which measurements were made from 10 oocytes per treatment. The asterisks denote a significant difference in drug uptake between the noninjected treatment and that measured in oocytes expressing a variant of PfCRT: *P < 0.05; **P < 0.01; ***P < 0.001 (one-way ANOVA).
(TIF)

S5 Fig Spermine and histidine do not inhibit the accumulation of [^3^H]CQ in oocytes expressing PfCRT. The uptake of [^3^H]CQ (0.25 μM) was measured in the absence (solvent control) or presence of the test compounds (extracellular concentrations of 1 and 2 mM). The assays were conducted at pH 5.5 and in the presence of 15 μM unlabeled CQ. The rates of CQ uptake (pmol per oocyte/h) in noninjected oocytes and PfCRT^Dd2^-expressing oocytes were 1.41 ± 0.02 and 24 ± 1.3, respectively. The data are the mean + SEM of four independent experiments performed using oocytes from different frogs), within which measurements were made from 10 oocytes per treatment. ‘ns’ denotes no significant difference from the PfCRT^Dd2^ control (*P* > 0.05; one-way ANOVA).
(TIF)

S6 Fig Amantadine *trans*-stimulates the uptake of [^3^H]CQ into oocytes expressing PfCRT in a concentration-dependent manner. Control oocytes (noninjected oocytes and oocytes expressing PfNT1) and oocytes expressing a variant of PfCRT (PfCRT^3D7^, 76K-PfCRT^K1^, PfCRT^K1^, 76I-PfCRT^K1^, or 76I,369F-PfCRT^K1^) were microinjected with buffer containing amantadine to achieve an estimated intracellular concentration ([amantadine]_i_) of 1 to 20 mM. A control was also performed in which the oocytes were microinjected with buffer alone. The rates of CQ uptake are the mean ± SEM of five independent experiments (performed using oocytes from different frogs), within which measurements were made from 10 oocytes per treatment. Where not shown, error bars fall within the symbols. The data presented in Fig 3E and 3F were calculated by subtracting the rate of CQ uptake measured in the buffer-injected control from that measured in each of the corresponding amantadine treatments (and within the same oocyte type). The noninjected data overlays the data obtained with oocytes expressing PfNT1, PfCRT^3D7^, or 76K-PfCRT^K1^.
(TIF)

S7 Fig Mechanisms for the CQ and quinidine susceptibilities conferred by PfCRT isoforms in the malaria parasite. (A) The variants of PfCRT^K1^ that contain 72R, 76K, 163R, 352K, or 352R (R/K) do not possess significant CQ transport activity. The drug would therefore remain in the DV where it exerts an anti-hemozoin effect that kills the parasite, which is consistent with the CQ-sensitive (S) status of the respective lines. By contrast, PfCRT^K1^ (76T) and 76I-PfCRT^K1^ transport CQ out of the parasite’s DV, thereby conferring CQ resistance (R). The addition of 369F to 76I-PfCRT^K1^ significantly reduces its affinity and capacity for CQ transport. Hence, 76I,369F-PfCRT^K1^ imparts a relatively low level of resistance (*low*-R) to CQ. (B) The R/K variants of PfCRT^K1^ do not possess significant quinidine (QD) transport activity whereas PfCRT^K1^, 76N-PfCRT^K1^ and 76I-PfCRT^K1^ each have the ability to transport QD out of the DV, albeit to varying degrees. These differences in QD transport activity explain, at least in part, the susceptibilities of the corresponding 106/1 parasite strains to QD. 76N-PfCRT^K1^ has a slightly lower affinity for QD then does PfCRT^K1^, and also has a much lower maximum rate of QD transport. The low capacity and low affinity of QD transport via 76N-PfCRT^K1^ explains why this protein has little net effect on the parasite’s sensitivity to QD. By contrast, the relatively high capacity of PfCRT^K1^ for QD transport is consistent with the decreased susceptibility of 106/1^76T^ parasites to QD (*low*-R). 76I-PfCRT^K1^ has a low maximum rate of QD transport, but this characteristic is counterbalanced by a 3-fold increase in its affinity for QD, and the net capacity of the protein for QD transport appears to be sufficient to reduce the parasite’s susceptibility to QD (at least under the conditions of the *in vitro* parasite proliferation assays).
(TIF)

S1 Table Primer sequences used to introduce mutations into the PfCRT coding sequence via site-directed mutagenesis. (DOCX)
