# Supplementary figures and images for: Molecular Mechanisms for Drug Hypersensitivity Induced by the Malaria Parasite’s Chloroquine Resistance Transporter
Source: PLoS Pathog. 2016 Jul 21;12(7):e1005725. doi: 10.1371/journal.ppat.1005725 (PMC4956231; doi:10.1371/journal.ppat.1005725)

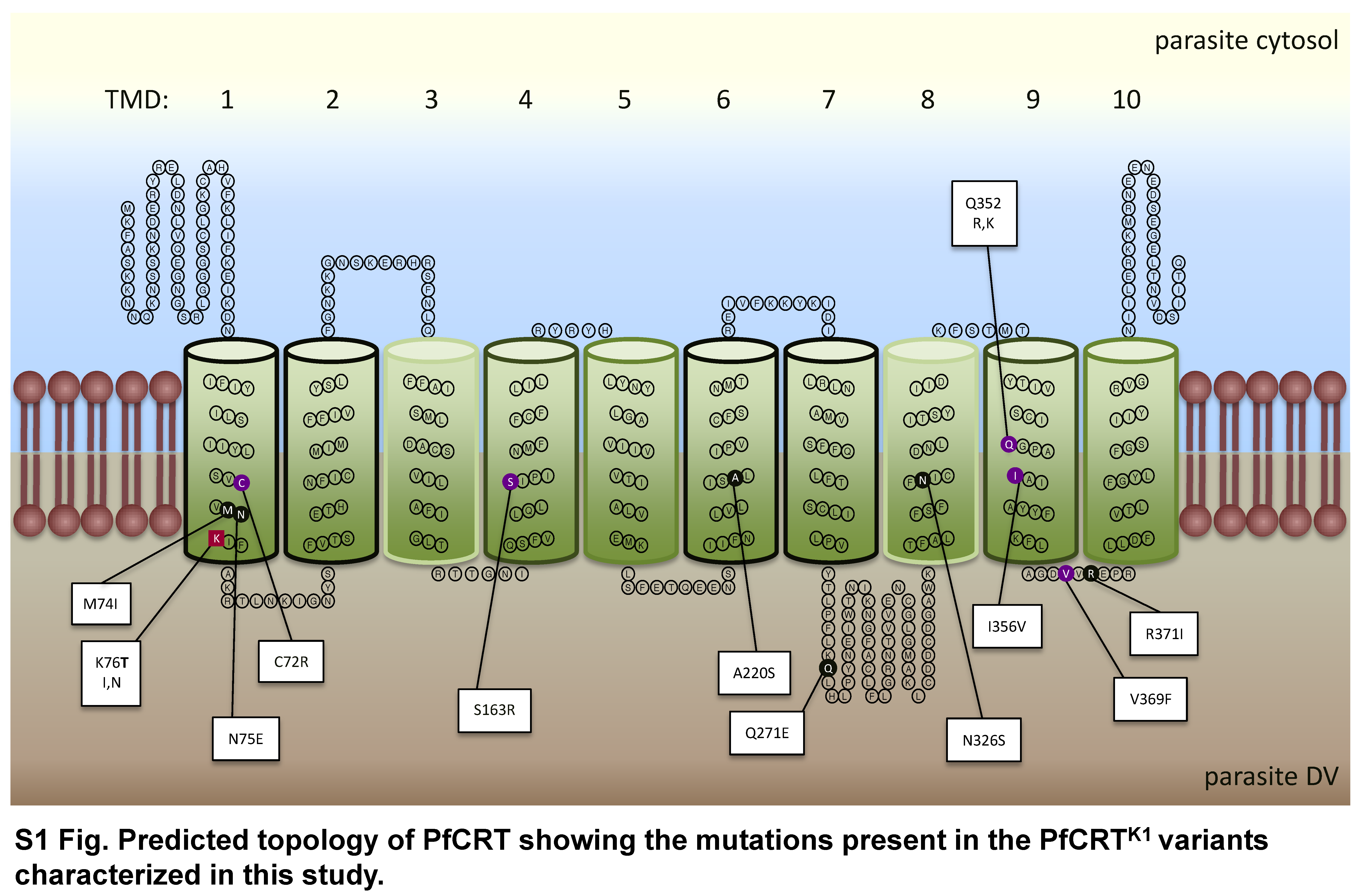

Supplement: S1 Fig — PfCRT is predicted to contain 10 α-helical transmembrane domains (TMDs) and to be orientated in the digestive vacuole (DV) membrane with the N- and C-termini extending into the parasite cytosol [83]. The positions of the mutated residues in PfCRTK1 are indicated with black circles. The key CQ resistance-associated mutation (K76T) is represented as a red square. The purple circles show the locations of the additional residues that are mutated in the variants of PfCRTK1. The box attached to each polymorphic residue lists the (non-wild-type) amino acid(s) that occur at that position. The predicted roles of the TMDs are as follows: 4 and 9 (outlined in dark green) are implicated in the binding and translocation of substrates, TMDs 3 and 8 (boxed in light green) are thought to assist in the binding and translocation of the substrate and may also influence the substrate-specificity of the transporter, TMDs 1, 2, 6, and 7 (boxed in black) may be involved in recognizing and discriminating between substrates, and TMDs 5 and 10 (outlined in mid-green) are thought to play a role in the formation of homo-dimers [83]. (TIFF) [file ppat.1005725.s001.tiff]

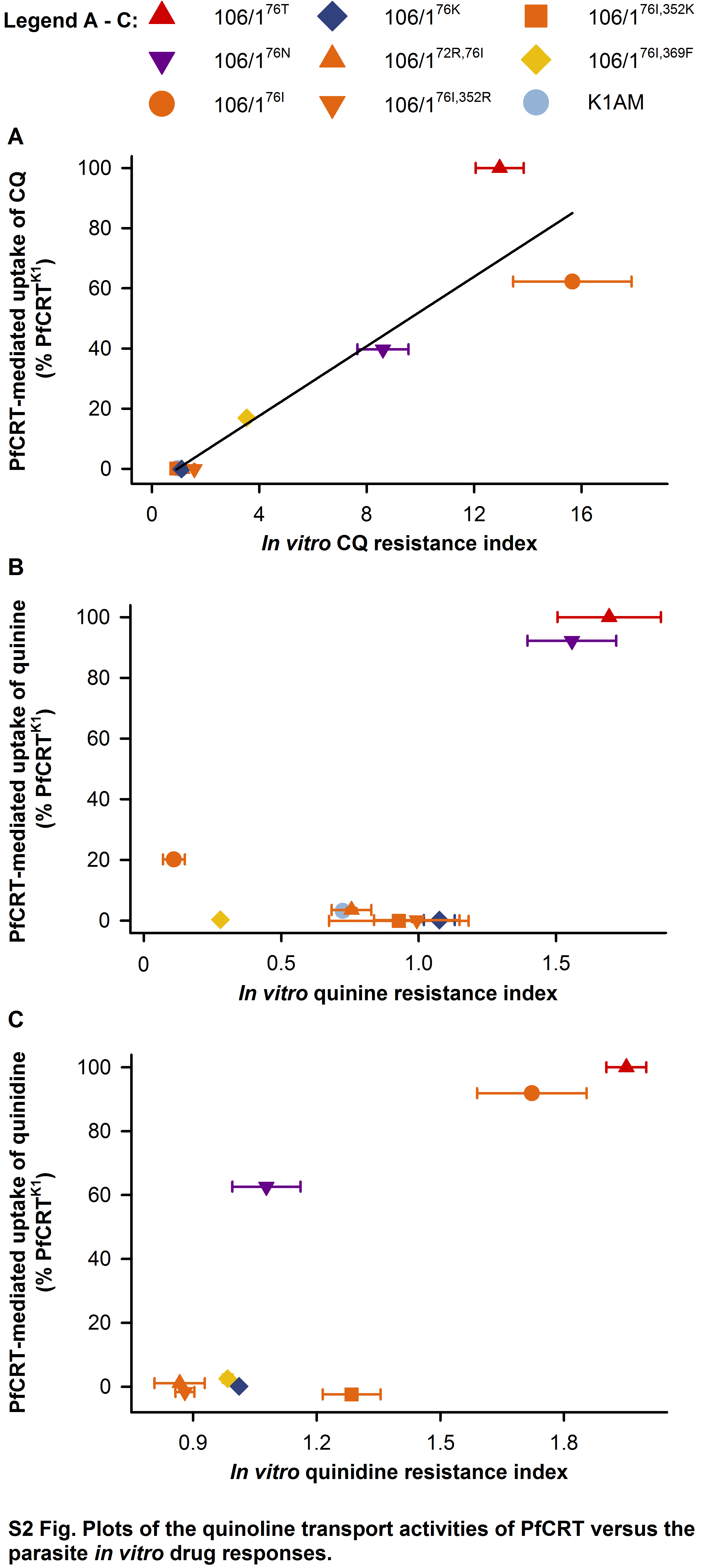

Supplement: S2 Fig — (A-C) The CQ, quinine, or quinidine transport activity of a given PfCRTK1 isoform (calculated from the data presented in Fig 2) was plotted against the in vitro resistance index for the relevant drug and parasite line (listed in Table 1). Where not shown, error bars fall within the symbols. There was a positive correlation between the in vitro response of a parasite line to CQ and the CQ transport properties of the corresponding isoform of PfCRTK1 (panel A: R2 = 0.856). This is consistent with our previous observation of a positive correlation (R2 = 0.858; [42]) in an analysis performed with seven field isoforms of PfCRT, but contrasts with the work of Roepe and colleagues, who have not detected a relationship between PfCRTCQR-mediated CQ transport and the parasite’s response to CQ [84, 85]. In panel B, the quinine transport activities of many of the PfCRTK1 isoforms correlated positively with the in vitro responses of the parasites to this drug. However, two key exceptions included the data for the highly QN-hypersensitive lines 106/176I and 106/176I,369F. Likewise, in panel C, a positive correlation between the capacity of a given PfCRTK1 isoform to transport quinidine and the relevant parasite’s in vitro quinidine resistance index held for all of the parasites bar the QD-sensitive line 106/176N. Refer to the main text for a discussion of these outlying points. Note also that other genetic elements, such as the amplification or mutation of the parasite’s multidrug resistance protein 1 (PfMDR1) and the altered expression of other genes, can contribute to the quinoline resistance phenotype [7, 86–90]. Hence, the CQ resistance index obtained for the 106/176I line, which is higher than what might be expected from the CQ transport activity of 76I-PfCRTK1, may be due to changes in the expression of one or more genes, including pfmdr1 [14, 89]. High-level CQ resistance has been associated with reductions in the pfmdr1 copy number and a corresponding decrease in the expre [file ppat.1005725.s002.TIF]

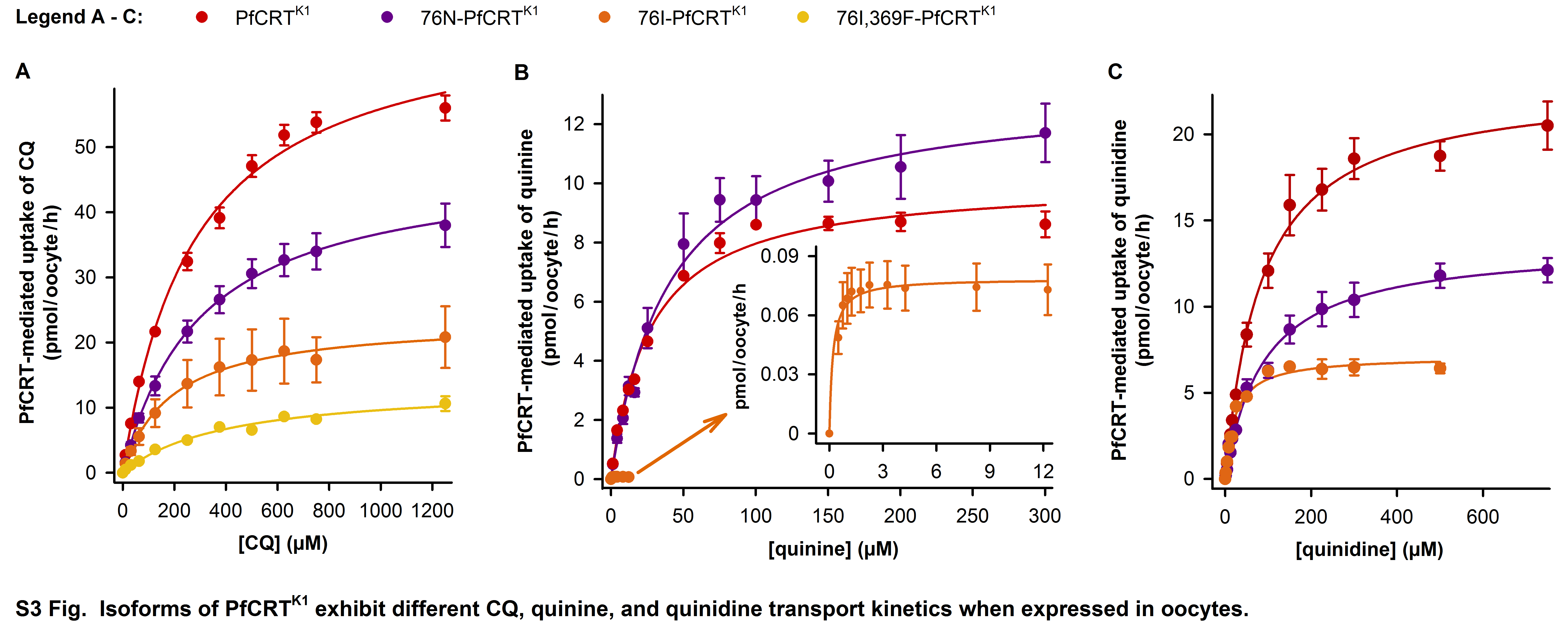

Supplement: S3 Fig — (A) The uptake of [3H]CQ (0.25 μM) was measured at pH 6.0 and over an extracellular concentration range of 10 to 1250 μM unlabeled CQ. (B) The uptake of [3H]quinine (0.25 μM) was measured at pH 5.0 and the extracellular concentration of unlabeled quinine ranged between 1 and 300 μM (PfCRTK1 and 76N-PfCRTK1) or 0.25 and 12 μM (76I-PfCRTK1). The inset shows a magnified plot of the 76I-PfCRTK1 data. (C) The uptake of [3H]quinidine (0.25 μM) was measured at pH 5.0 and the extracellular concentration of unlabeled quinidine ranged between 1 and 500 μM (PfCRTK1 and 76I-PfCRTK1) or 1 and 750 μM (76N-PfCRTK1). In all cases, the rate of PfCRT-mediated drug uptake was calculated by subtracting the rate measured in oocytes expressing 76K-PfCRTK1 from that measured in oocytes expressing PfCRTK1, 76N-PfCRTK1, 76I-PfCRTK1, or 76I,369F-PfCRTK1 at each drug concentration. The Michaelis-Menten equation was fitted to the resulting data using nonlinear regression. The rates of drug uptake are the mean ± SEM of multiple independent experiments (performed using oocytes from different frogs), within which measurements were made from 10 oocytes per treatment. Where not shown, error bars fall within the symbols. The exact n values, as well as the kinetic parameters derived from these data, are presented in Table 2. (TIF) [file ppat.1005725.s003.TIF]

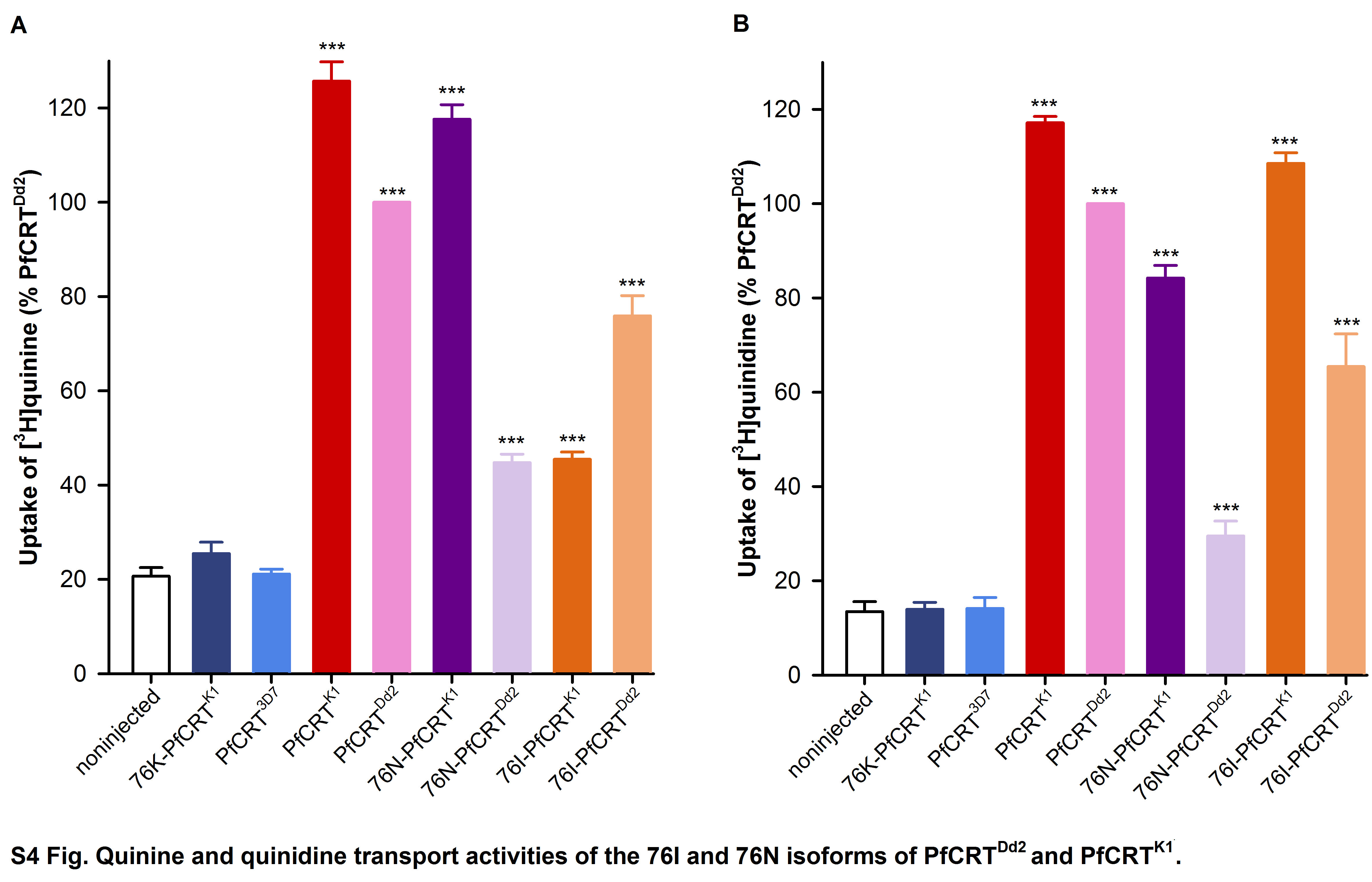

Supplement: S4 Fig — Noninjected oocytes accumulate low levels of quinine and quinidine via simple diffusion of the neutral species of the drug [29]. This represents the background level of drug accumulation. The uptake of (A) [3H]quinine and (B) [3H]quinidine was measured at pH 5.0 and in the presence of 1 μM of the respective unlabeled drug. The concentration of the [3H]drug was 0.25 μM. The rates of uptake (pmol per oocyte/h) in noninjected oocytes and oocytes expressing PfCRTDd2 were 0.10 ± 0.01 and 0.44 ± 0.04, respectively, for quinine and 0.05 ± 0.01 and 0.30 ± 0.07, respectively, for quinidine. In both panels, drug uptake is expressed relative to that measured in oocytes expressing PfCRTDd2. The rates of quinine and quinidine uptake mediated by PfCRTK1 were 1.2–1.3 times that measured for PfCRTDd2, whereas the 76N-PfCRTK1 protein possessed 3.1 times the quinine transport activity, and 3.2 times the quinidine transport activity, of 76N-PfCRTDd2. Moreover, the rate of quinidine transport mediated by 76I-PfCRTK1 was 1.5 times that measured for 76I-PfCRTDd2. The single exception to this trend was the rate of quinine transport via 76I-PfCRTK1, which was 0.55 times that measured for its PfCRTDd2 counterpart. The data are the mean + SEM of at least five independent experiments (performed using oocytes from different frogs), within which measurements were made from 10 oocytes per treatment. The asterisks denote a significant difference in drug uptake between the noninjected treatment and that measured in oocytes expressing a variant of PfCRT: *P < 0.05; **P < 0.01; ***P < 0.001 (one-way ANOVA). (TIF) [file ppat.1005725.s004.TIF]

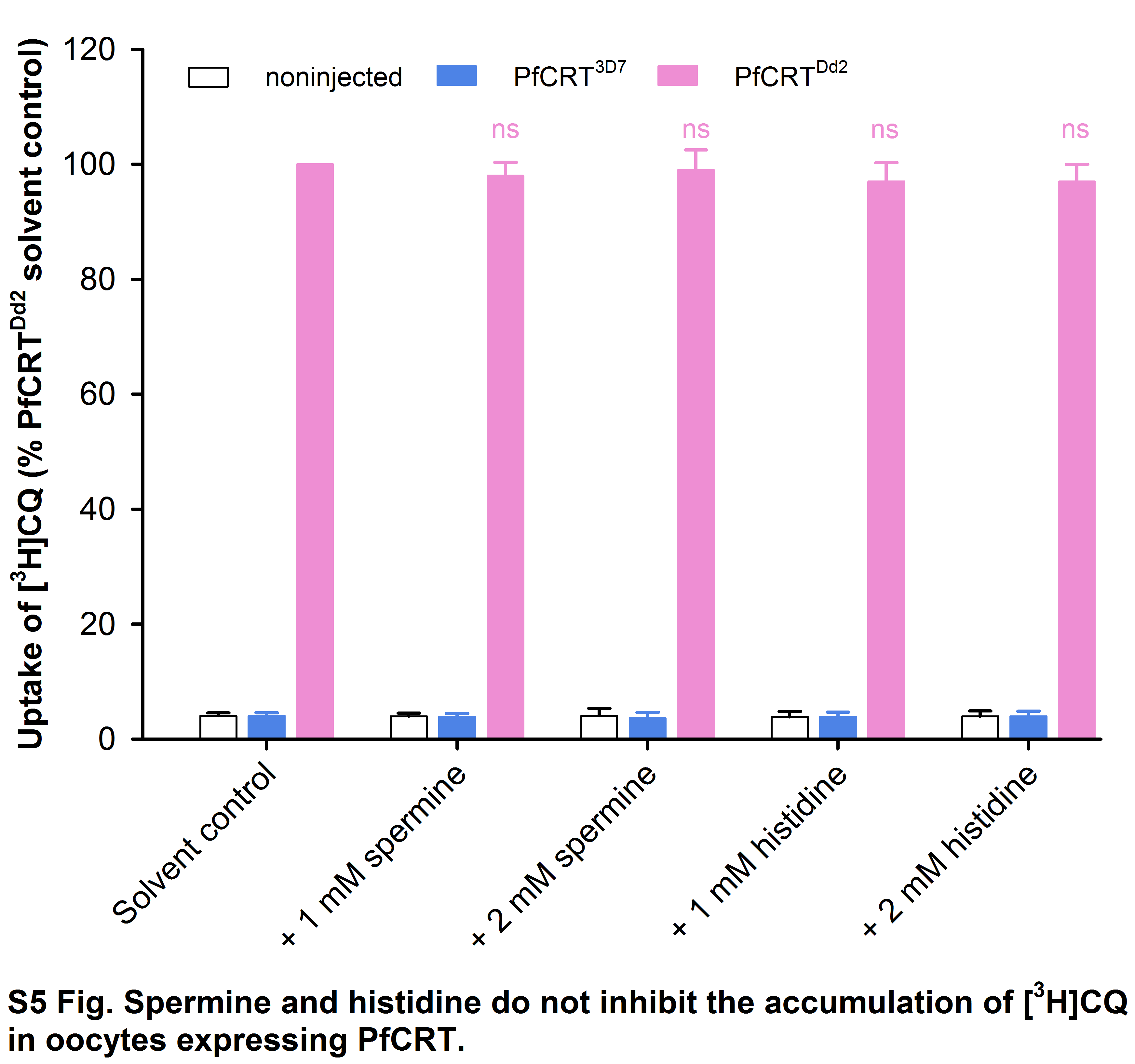

Supplement: S5 Fig — The uptake of [3H]CQ (0.25 μM) was measured in the absence (solvent control) or presence of the test compounds (extracellular concentrations of 1 and 2 mM). The assays were conducted at pH 5.5 and in the presence of 15 μM unlabeled CQ. The rates of CQ uptake (pmol per oocyte/h) in noninjected oocytes and PfCRTDd2-expressing oocytes were 1.41 ± 0.02 and 24 ± 1.3, respectively. The data are the mean + SEM of four independent experiments performed using oocytes from different frogs), within which measurements were made from 10 oocytes per treatment. ‘ns’ denotes no significant difference from the PfCRTDd2 control (P > 0.05; one-way ANOVA). (TIF) [file ppat.1005725.s005.TIF]

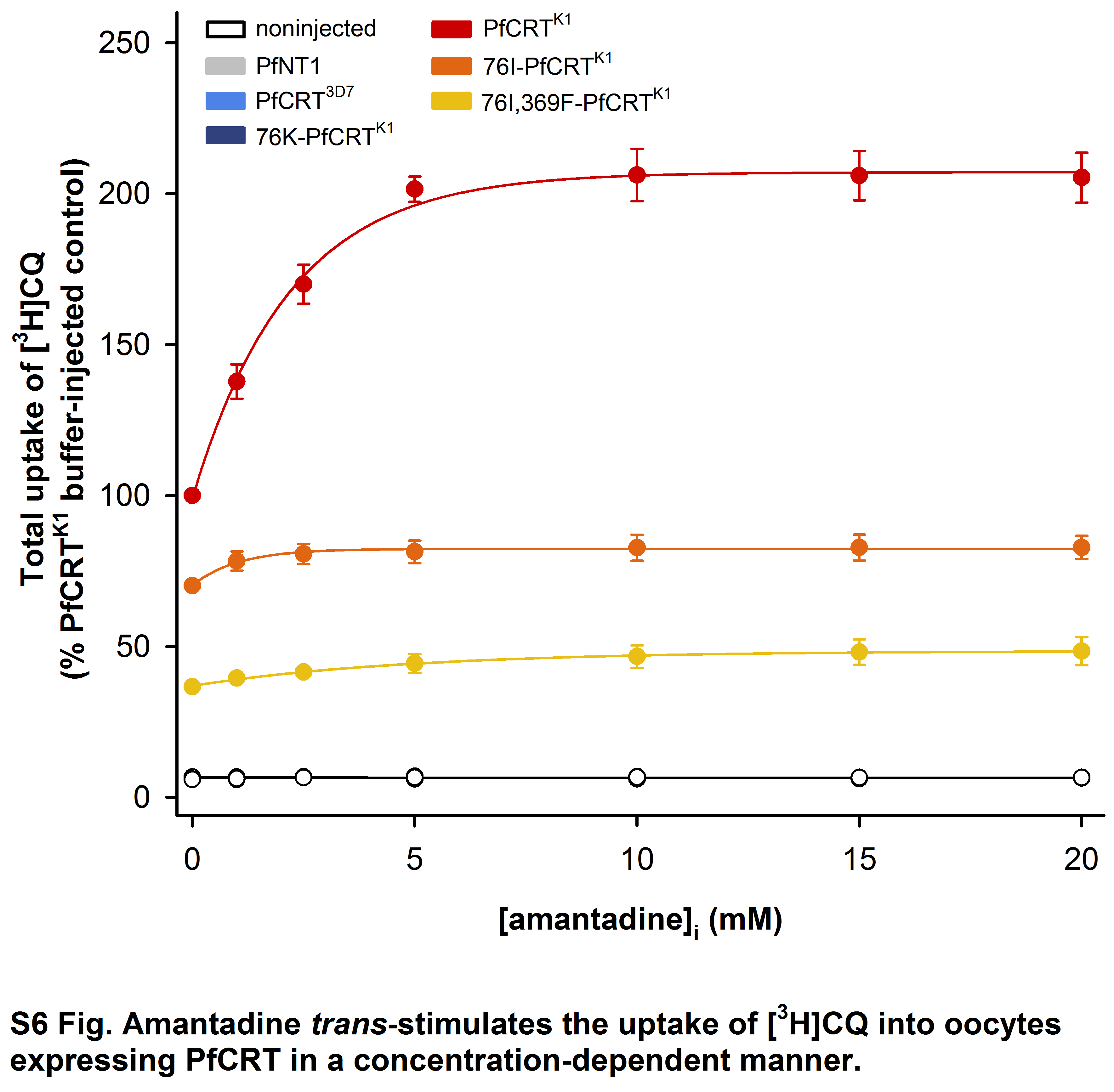

Supplement: S6 Fig — Control oocytes (noninjected oocytes and oocytes expressing PfNT1) and oocytes expressing a variant of PfCRT (PfCRT3D7, 76K-PfCRTK1, PfCRTK1, 76I-PfCRTK1, or 76I,369F-PfCRTK1) were microinjected with buffer containing amantadine to achieve an estimated intracellular concentration ([amantadine]i) of 1 to 20 mM. A control was also performed in which the oocytes were microinjected with buffer alone. The rates of CQ uptake are the mean ± SEM of five independent experiments (performed using oocytes from different frogs), within which measurements were made from 10 oocytes per treatment. Where not shown, error bars fall within the symbols. The data presented in Fig 3E and 3F were calculated by subtracting the rate of CQ uptake measured in the buffer-injected control from that measured in each of the corresponding amantadine treatments (and within the same oocyte type). The noninjected data overlays the data obtained with oocytes expressing PfNT1, PfCRT3D7, or 76K-PfCRTK1. (TIF) [file ppat.1005725.s006.TIF]

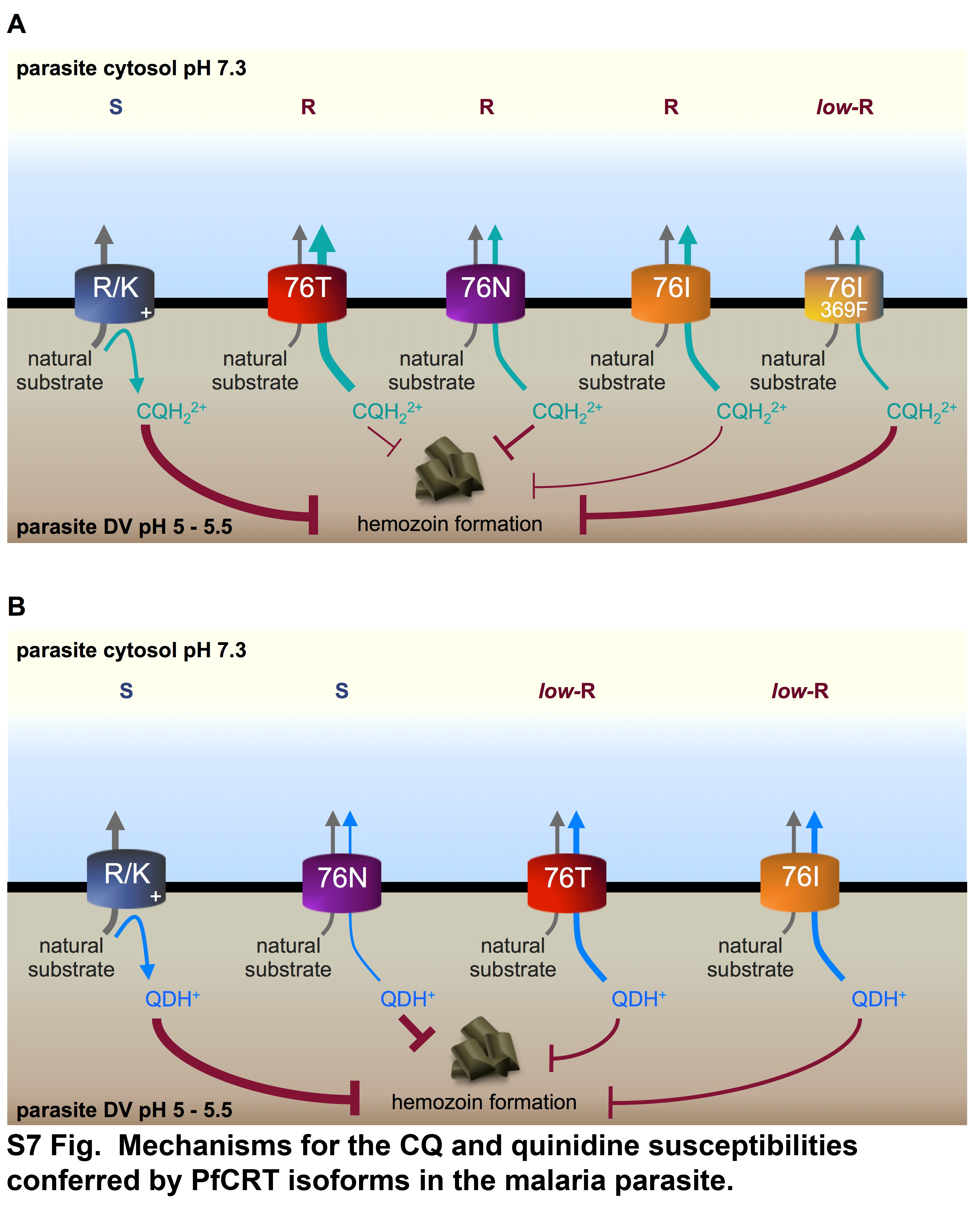

Supplement: S7 Fig — (A) The variants of PfCRTK1 that contain 72R, 76K, 163R, 352K, or 352R (R/K) do not possess significant CQ transport activity. The drug would therefore remain in the DV where it exerts an anti-hemozoin effect that kills the parasite, which is consistent with the CQ-sensitive (S) status of the respective lines. By contrast, PfCRTK1 (76T) and 76I-PfCRTK1 transport CQ out of the parasite’s DV, thereby conferring CQ resistance (R). The addition of 369F to 76I-PfCRTK1 significantly reduces its affinity and capacity for CQ transport. Hence, 76I,369F-PfCRTK1 imparts a relatively low level of resistance (low-R) to CQ. (B) The R/K variants of PfCRTK1 do not possess significant quinidine (QD) transport activity whereas PfCRTK1, 76N-PfCRTK1 and 76I-PfCRTK1 each have the ability to transport QD out of the DV, albeit to varying degrees. These differences in QD transport activity explain, at least in part, the susceptibilities of the corresponding 106/1 parasite strains to QD. 76N-PfCRTK1 has a slightly lower affinity for QD then does PfCRTK1, and also has a much lower maximum rate of QD transport. The low capacity and low affinity of QD transport via 76N-PfCRTK1 explains why this protein has little net effect on the parasite’s sensitivity to QD. By contrast, the relatively high capacity of PfCRTK1 for QD transport is consistent with the decreased susceptibility of 106/176T parasites to QD (low-R). 76I-PfCRTK1 has a low maximum rate of QD transport, but this characteristic is counterbalanced by a 3-fold increase in its affinity for QD, and the net capacity of the protein for QD transport appears to be sufficient to reduce the parasite’s susceptibility to QD (at least under the conditions of the in vitro parasite proliferation assays). (TIF) [file ppat.1005725.s007.tif]
