# Supplementary material for: Molecular Mechanisms for Drug Hypersensitivity Induced by the Malaria Parasite’s Chloroquine Resistance Transporter
Source: PLoS Pathog. 2016 Jul 21;12(7):e1005725. doi: 10.1371/journal.ppat.1005725 (PMC4956231; doi:10.1371/journal.ppat.1005725)
Supplement: S1 Table — (DOCX) [file ppat.1005725.s008.docx]

**S1 Table. Primer sequences used to introduce mutations into the PfCRT coding sequence via site-directed mutagenesis.**

| **Template** | **Mutation** | **Forward PCR primer 5’ -> 3’**^a^ | **Reverse PCR primer 5’ -> 3’**^a^ | **Resulting construct** |
| --- | --- | --- | --- | --- |
| ^b^PfCRT^K1^ | T76K | GTTGTGTCATTGAAaagATTTTTGCGAAGAG | CTCTTCGCAAAAATcttTTCAATGACACAAAC | 76K-PfCRT^K1^ |
| PfCRT^K1^ | T76N | GTTTGTGTCATTGAAaatATTTTTGCGAGAAC | GTTCTCTTCGCAAAAATattTTC | 76N-PfCRT^K1^ |
| PfCRT^K1^ | T76I | GTTTGTGTCATTGAAattATTTTTGCGAAGAGAAC | CTCTTCGCAAAAATaatTTCAATGACACAAAC | 76I-PfCRT^K1^ |
| 76I-PfCRT^K1^ | C72R | CTACCTGTCTGTTagaGTCATTGAAATT | AATTTCAATGACtctAACAGACAGGTAG | 72R,76I-PfCRT^K1^ |
| 76I-PfCRT^K1^ | Q352K | GATTGTTTCTTGTATCaaaGGGCCCGCC | GGCGGGCCCtttGATACAAGAAACAATC | 76I,352K-PfCRT^K1^ |
| 76I-PfCRT^K1^ | Q352R | GTTTCTTGTATCcgaGGGCCCGC | GTGGCGGGCCCtcgGATACAAG | 76I,352R-PfCRT^K1^ |
| 76I-PfCRT^K1^ | V369F | CGAttcGTGATTGAAC | TCACgaaATCGCCCGCCAG | 76I,369F-PfCRT^K1^ |
| PfCRT^K1^ | S163R | GCTGagaATTCCCATAAC | GGGAATtctCAGCTGTAGAAC | 163R-PfCRT^K1^ |
| 163R-PfCRT^K1^ | T356V | GCCgtgGCGATCGCGTATTACTTCAAATTCC | GTAATACGCGATCGCcacGGCGGGCCCC | 163R,356V-PfCRT^K1^ |

^a^Mutated codons are shown in lowercase.

^b^The PCR template was a codon-harmonized version of the PfCRT^K1^ sequence that encodes a retention motif-free form of the protein, and which therefore localizes to the oocyte plasma membrane [[30](#_ENREF_1), [42](#_ENREF_2)].
